# Targeting the ferritinophagy-lysosome axis as a therapeutic vulnerability in gastroenteropancreatic neuroendocrine tumors

**DOI:** 10.1016/j.xcrm.2026.102695

**Published:** 2026-03-24

**Authors:** Yizhi Cao, Caleb Cheng, Yitong Yin, Sarah N. Yee, Yang Zheng, Somnath Mahapatra, Radha Paturu, Andrej Coleski, Shannon VanAken, Fan Yang, Rüya Pakkan, Yi Zhao, Rupam Bhattacharyya, Stephanie J. Miner, Xuhong Cao, Rahul Mannan, Chungen Li, Vaibhav Sahai, Ke Ding, Costas A. Lyssiotis, Arul M. Chinnaiyan, Yuanyuan Qiao

**Affiliations:** 1 Michigan Center for Translational Pathology, University of Michigan, Ann Arbor, MI, USA; 2 Department of General Surgery, Pancreatic Disease Center, Ruijin Hospital, Shanghai Jiao Tong University School of Medicine, Shanghai, P.R. China; 3 Cellular and Molecular Biology Program, University of Michigan, Ann Arbor, MI, USA; 4 State Key Laboratory of Chemical Biology, Shanghai Institute of Organic Chemistry, Chinese Academy of Sciences, Shanghai, P.R. China; 5 Rogel Cancer Center, University of Michigan, Ann Arbor, MI, USA; 6 Division of Hematology and Oncology, Department of Internal Medicine, University of Michigan, Ann Arbor, MI, USA; 7 Department of Molecular and Integrative Physiology, University of Michigan, Ann Arbor, MI, USA; 8 Department of Internal Medicine, Division of Gastroenterology, University of Michigan, Ann Arbor, MI, USA; 9 Department of Pathology, University of Michigan, Ann Arbor, MI, USA; 10 Howard Hughes Medical Institute, University of Michigan, Ann Arbor, MI, USA; 11 Department of Urology, University of Michigan, Ann Arbor, MI, USA

**Keywords:** lysosome, lipid metabolism, mTOR, ferritinophagy, gastroenteropancreatic neuroendocrine tumor, PIKfyve

## Abstract

mTOR inhibitors (mTORis) are Food and Drug Administration (FDA)-approved therapies for advanced gastroenteropancreatic neuroendocrine tumors (GEP-NETs), yet their clinical efficacy is often limited by transient responses and acquired resistance. To uncover sensitizing co-targets, we conduct a kinome-wide CRISPR-Cas9 screen, identifying the lipid kinase PIKfyve as a key vulnerability in GEP-NETs. PIKfyve is overexpressed and functionally linked to the regulation of lipid biosynthesis through the mTOR-SREBP1 axis. Mechanistically, PIKfyve inhibition impairs lysosome-mediated ferritin degradation, amplifying metabolic stress triggered by mTORi-induced ferritinophagy. Co-inhibition of mTOR and PIKfyve synergistically disrupts lipid and iron metabolism, leading to enhanced tumor suppression and improved survival in preclinical GEP-NET models. These findings nominate PIKfyve as a metabolic co-target to overcome mTORi resistance, offering a rationale for combination therapies in mTOR-driven malignancies.

## Highlights

• CRISPR screen identifies PIKfyve as a metabolic vulnerability in GEP-NETs
• PIKfyve regulates lipid biosynthesis via mTOR-SREBP1 and maintains lysosomal iron flux
• mTOR inhibition induces NCOA4-dependent ferritinophagy
• Co-inhibition of mTOR and PIKfyve perturbs lipid and iron homeostasis

## Graphical abstract

**Figure:**
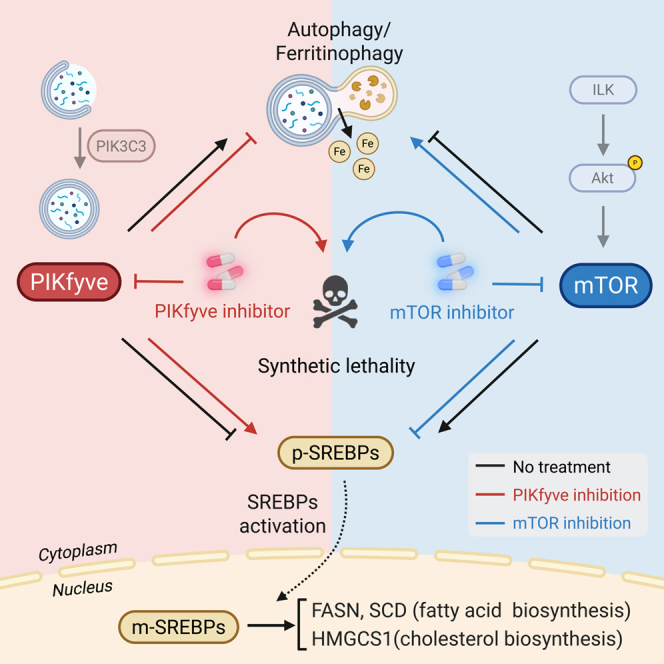

## Teaser

Cao et al. demonstrate synthetic lethality between PIKfyve and mTOR signaling in GEP-NETs. PIKfyve sustains ferritinophagy and limits lipid synthesis, whereas mTOR promotes lipogenesis and suppresses ferritinophagy. Dual inhibition disrupts lysosomal degradation, lipid metabolism, and iron metabolism, causing metabolic collapse and tumor cell death—a potential therapeutic strategy for GEP-NETs.

## Introduction

Gastroenteropancreatic neuroendocrine tumors (GEP-NETs) are rare malignancies arising from neuroendocrine cells in the gastrointestinal tract or pancreas and account for around 70% of all neuroendocrine tumors.^1^ Although uncommon, GEP-NET incidence has increased markedly over the past four decades.^2^ High-grade and metastatic GEP-NETs have poor prognosis, with 5-year survival rates as low as 37.6%.^3^ Current standard-of-care targeted therapy relies on mammalian target of rapamycin (mTOR) inhibition with everolimus^4^^,^^5^; however, responses are often transient, and resistance frequently develops.^6^

mTOR forms two complexes (mTORC1 and mTORC2) with distinct substrates and cellular functions.^7^ mTORC1 is the master regulator of protein, lipid, nucleotide, and ATP production and suppresses autophagy to support cell growth. mTORC1 promotes protein synthesis via phosphorylation of 4E-BPs and p70S6 kinase and drives lipid synthesis through sterol regulatory element-binding proteins (SREBP1/2). Under low sterol conditions, SREBPs are proteolytically activated and translocate to the nucleus to induce lipid and cholesterol synthesis genes.^8^^,^^9^ mTORC2 phosphorylates AKT to promote cell growth.^7^ mTORC1 signaling is elevated in over 50% of human cancers^10^; however, mTORC2 activity is less defined in cancers. The mTOR inhibitor, everolimus, binds to FKBP12 and directly inhibits mTORC1 with limited effects on mTORC2.^11^ Everolimus is approved for the treatment of GEP-NETs,^12^ renal cell carcinomas,^13^ subependymal giant cell astrocytomas,^14^ and breast cancer.^15^ However, mTOR inhibitors are largely cytostatic,^7^ as mTOR inhibition induces autophagy, a key resistance mechanism in nutrient-limited tumors.^16^ Torin-1 is an ATP-competitive inhibitor targeting both mTORC1 and mTORC2, though its clinical utility remains untested.^17^

PIKfyve is a phosphoinositide 5-kinase that generates phosphatidylinositol 3,5-bisphosphate [PI(3,5)P_2_] from PI3P on endosomes and lysosomes.^18^ It regulates autophagy by promoting autophagosome-lysosome fusion, and its inhibition disrupts autophagic flux and causes lysosomal enlargement *in vitro* and *in vivo*. PIKfyve has emerged as a therapeutic target in several malignancies, including prostate cancer, pancreatic ductal adenocarcinoma (PDAC), multiple myeloma, and breast cancer.^18^^,^^19^^,^^20^^,^^21^ We previously showed that PIKfyve is upregulated in PDAC tumors, and that *Pikfyve* knockout prolongs survival and limits disease progression in a Kras-driven mouse model.^21^ Targeting of autophagy has not been successful in patients thus far, as the current therapeutic agent, hydroxychloroquine (HCQ), failed to achieve effective autophagy blockade in patients.^22^^,^^23^^,^^24^ Collective evidence supports PIKfyve as a promising therapeutic target in the autophagy pathway, warranting investigation in GEP-NETs.

In this study, we identify PIKfyve as a previously unrecognized therapeutic target in GEP-NETs using an unbiased CRISPR screen and show that PIKfyve is overexpressed in malignant tissue. Mechanistically, GEP-NETs depend on PIKfyve-mediated lipid homeostasis, which is regulated by mTOR signaling and suppressed by mTOR inhibitors such as everolimus. As a standard-of-care therapy, mTOR inhibition induces ferritinophagy and relies on PIKfyve-dependent autophagy. Together, this study identifies a synthetic lethal interaction between PIKfyve and mTOR inhibition in GEP-NETs and supports combined targeting of these pathways in GEP-NETs and other mTOR-driven cancers.

## Results

### Kinome-wide CRISPR knockout screen reveals the VPS34-PIKfyve pathway as a druggable target in GEP-NETs

**Figure 1:**
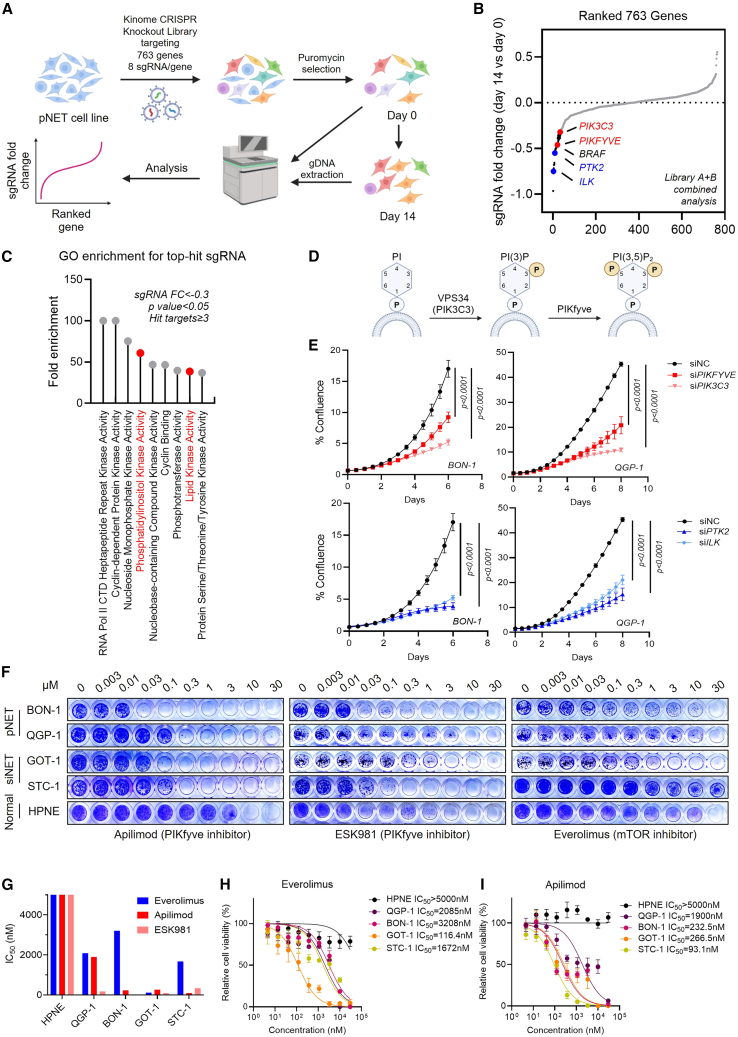
Kinome-wide CRISPR knockout screen identifies PIKfyve as a druggable target in GEP-NETs (A) Schematic of the CRISPR screening workflow in BON-1-DsRed-IRES-GFP-p62 cells. (B) Gene enrichment rank plot from kinome CRISPR knockout screens (specified in Table S1). Cutoff was set at fold change < −0.3. Red genes are related to the phosphatidylinositol metabolic pathway, while blue genes are related to the mTOR pathway. (C) Pathway enrichment analysis of essential genes from CRISPR screening data in (B) using PANTHER 19.0. Targets with a fold change < −0.3 were considered candidate genes, and pathways with a *p* value <0.05 and hit targets ≥3 were displayed in the plot. Pathways labeled in red are related to phosphatidylinositol metabolism. (D) Schematic of PI(3,5)P2 biosynthesis regulated by VPS34 (*PIK3C3*) and PIKfyve. (E) Cell confluence of BON-1 and QGP-1 cells after siRNA-mediated knockdown of *PIK3C3*, *PIKFYVE*, or non-targeting control (siNC). Data are presented as mean ± SD (*n* = 4 biological replicates). Two-way ANOVA. (F) Crystal violet staining showing long-term growth inhibition by PIKfyve inhibitor apilimod and mTOR inhibitor (Torin-1 or everolimus) in indicated GEP-NETs cell lines and HPNE cells. (G) Compilation of IC_50_ values for everolimus, apilimod, and ESK981 in GEP-NET and HPNE cells. (H and I) Dose-response proliferation curves of everolimus (H) and apilimod (I) in indicated GEP-NET cell lines and HPNE cells. Data are presented as mean ± SD (at least three biological replicates).

To identify therapeutic targets in GEP-NETs, we employed a kinome-wide CRISPR knockout screen targeting 763 kinases in a pancreatic neuroendocrine tumor (pNET) cell line, BON-1 (Figure 1A). Integrated analysis of library A (single guide RNA [sgRNA] 1–4) and B (sgRNA 5–8) revealed four genes, including *ILK*, *PTK2* (*FAK*), *PIKFYVE*, and *PIK3C3* (*VPS34*), that were critical for pNET survival but not pan-essential (Figures 1B and S1A). *PIKFYVE* and *ILK* were commonly prioritized in both libraries, confirming the selective essentiality of these targets in pNET (Figure S1B). Gene Ontology (GO) enrichment of the top altered sgRNAs highlighted phosphatidylinositol (PI) kinase activity and lipid kinase activity pathways (Figure 1C). *PIK3C3* (*VPS34*) and *PIKFYVE* are lipid kinases from the same pathway controlling PI3P to PI(3,5)P_2_ biosynthesis^25^ (Figure 1D), further suggesting that the VPS34-PIKfyve pathway plays a critical role in pNET survival. Notably, *PTK2 (FAK*) and *ILK* were previously reported as upstream regulators of the mTOR pathway,^26^^,^^27^^,^^28^ suggesting the involvement of mTOR signaling and confirming the use of mTOR-targeted therapy in pNETs.^12^

The CRISPR screen results were next validated by small interfering RNA (siRNA) knockdown of *PIK3C3*, *PIKFYVE*, *PTK2*, and *ILK* in additional pNET cell lines, QGP-1 and BON-1. The knockdown efficiency of these targets was confirmed by qPCR (Figures S1C and S1D). Real-time cell proliferation assays showed that knockdown of each kinase significantly reduced cell growth (Figure 1E). Additionally, pronounced cytoplasmic vacuolization was observed only in *PIKFYVE* or *PIK3C3* knockdown conditions, consistent with prior reports in prostate cancer and PDAC^18^^,^^21^^,^^29^ (Figure S1E).

We next examined pharmacologic inhibition of the PIK3C3-PIKfyve pathway in GEP-NETs using apilimod or ESK981 (PIKfyve inhibitors) and SAR405 (PIK3C3 inhibitor). Results from long-term survival assays indicated that PIKfyve inhibition led to enhanced inhibition of cellular proliferation compared to PIK3C3 inhibition, consistent with PIKfyve acting downstream. These results were similar between apilimod and ESK981 treatments (Figures 1F and S1F). Importantly, pNETs (QGP-1 and BON-1) and small intestinal NETs (siNETs) (GOT-1 and STC-1) showed uniform sensitivity to PIKfyve inhibitors apilimod and ESK981, whereas normal pancreatic HPNE cells were resistant (Figure 1G). IC_50_ comparisons of apilimod, ESK981, PIK5-33d (a PIKfyve PROTAC degrader),^21^ and everolimus showed that PIKfyve inhibition induced greater anti-proliferative effects than the standard-of-care mTOR inhibitor, everolimus (Figures 1F–1I, S1G, and S1H). Together, our results establish PIKfyve as an essential survival factor and a previously unrecognized therapeutic target in GEP-NETs.

### PIKfyve is overexpressed and serves as a therapeutic target in GEP-NETs

**Figure 2:**
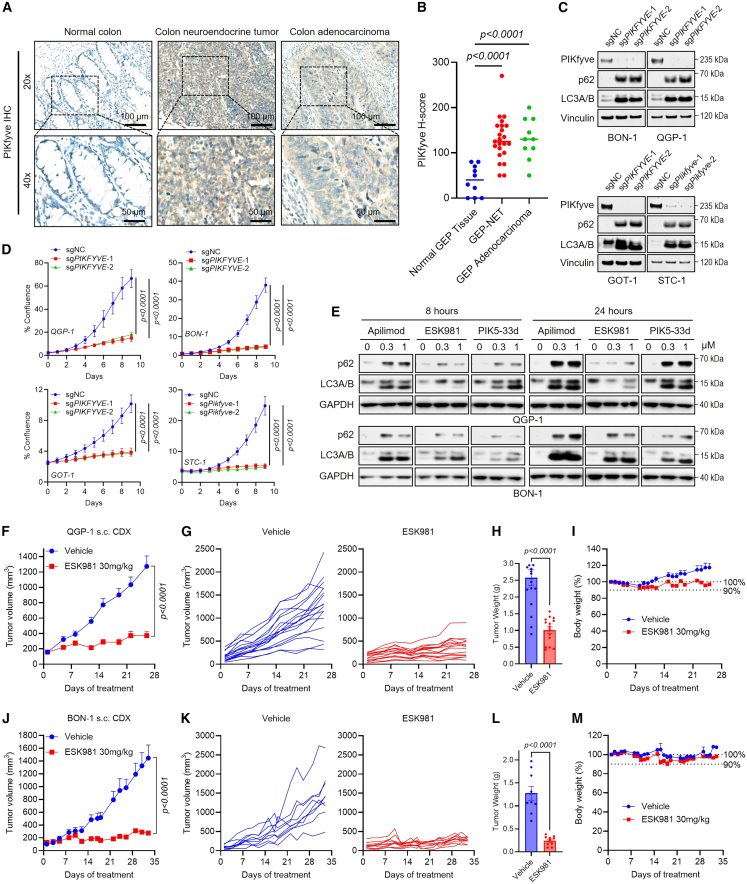
PIKfyve is overexpressed in GEP-NETs and serves as a therapeutic target (A) Representative PIKfyve IHC staining in human normal colon tissue, colon neuroendocrine tumor, and colon adenocarcinoma samples. (B) Quantification of PIKfyve H-score from tissue microarray of human normal GEP tissue, GEP-NETs, and GEP adenocarcinoma (specified in Table S2). Statistics were performed using one-way ANOVA. (C) Immunoblot analysis of PIKfyve and autophagy markers (p62 and LC3A/B) in GEP-NET cell lines following CRISPRi-mediated *PIKFYVE* knockdown. Vinculin served as the loading control. (D) Cell confluence of GEP-NET cell lines with CRISPRi-mediated *PIKFYVE* (sg*PIKFYVE* or sg*Pikfyve*) knockdown or control (sgNC). Data are presented as mean ± SD (*n* = 4 biological replicates). Two-way ANOVA. (E) Immunoblot analysis of autophagy markers in QGP-1 and BON-1 cells following PIKfyve inhibitors (apilimod or ESK981) or PIKfyve degrader (PIK5-33d) treatment for 8 or 24 h. GAPDH was used as a loading control. (F) Average tumor volumes of QGP-1 subcutaneous-cell-line-derived (CDX) model for vehicle (*n* = 9) or ESK981 (30 mg/kg, *n* = 10) treatment. Mean ± SEM. Two-way ANOVA. s.c., subcutaneous. (G) Spider plot displaying individual tumor volumes from vehicle or ESK981 treatment groups in QGP-1 subcutaneous CDX study. (H) Individual tumor weights of QGP-1 subcutaneous CDX model tumors at study endpoint. Unpaired two-tailed *t* test. (I) Percent body weight change of QGP-1 subcutaneous CDX model tumors from vehicle or ESK981 treatment groups. (J) Average tumor volumes of BON-1 subcutaneous CDX model following vehicle (*n* = 17) or ESK981 (30 mg/kg, *n* = 15) treatment. Mean ± SEM. Two-way ANOVA. (K) Spider plot displaying individual tumor volumes from vehicle or ESK981 treatment groups in BON-1 subcutaneous CDX study. (L) Individual tumor weights of BON-1 subcutaneous CDX model tumors at study endpoint. Unpaired two-tailed *t* test. (M) Percent body weight change of BON-1 subcutaneous CDX model tumors from vehicle or ESK981 treatment groups.

The role of PIKfyve in the development of GEP-NETs is unexplored. Here, we determined its expression level on a patient tissue microarray (TMA) consisting of 10 normal tissues, 23 neuroendocrine tumors, and 10 adenocarcinomas. A validated PIKfyve antibody (Figure S1I) was used for PIKfyve immunohistochemistry (IHC) staining. PIKfyve H-score analysis demonstrated that PIKfyve protein expression was similar in neuroendocrine tumor and adenocarcinoma tissues and both exhibited significantly and consistently higher PIKfyve expression levels than normal tissues (Figures 2A and 2B). This marked overexpression suggests that PIKfyve-driven processes may contribute to GEP-NET pathogenesis.

We next generated stable CRISPR interference (CRISPRi)-based *PIKFYVE* knockdown cell lines in human pNET (QGP-1, BON-1), human siNET (GOT-1), and murine siNET (STC-1) models. Genetic knockdown of *PIKFYVE* markedly reduced PIKfyve protein and impaired autophagic flux evidenced by increased lipidated LC3A/B and p62 levels (Figure 2C). Proliferation assays confirmed that PIKfyve depletion significantly inhibited GEP-NET growth (Figure 2D). Pharmacologic inhibition with apilimod, ESK981, or PIK5-33d similarly blocked autophagy (Figures 2E and S1J) and induced cytoplasmic vacuolization, a hallmark of PIKfyve inhibition (Figure S1K). These results confirm the functional importance of PIKfyve in maintaining autophagic flux and survival in GEP-NETs.

Furthermore, we evaluated the *in vivo* anti-tumor efficacy of PIKfyve inhibition using ESK981, a phase II investigational PIKfyve inhibitor, in QGP-1 and BON-1 subcutaneous-cell-line-derived xenograft (CDX) models.^30^^,^^31^ ESK981 markedly and consistently reduced tumor volume and weight compared with the vehicle group (Figures 2F–2H, 2J–2L, S1L, and S1M), while host body weights remained unaffected, indicating the treatment was well tolerated (Figures 2I and 2M). Taken together, these findings establish PIKfyve as a critical regulator of autophagic flux and cell proliferation in GEP-NETs that can be therapeutically targeted to control tumor growth.

### PIKfyve mediates lipid homeostasis in GEP-NETs

**Figure 3:**
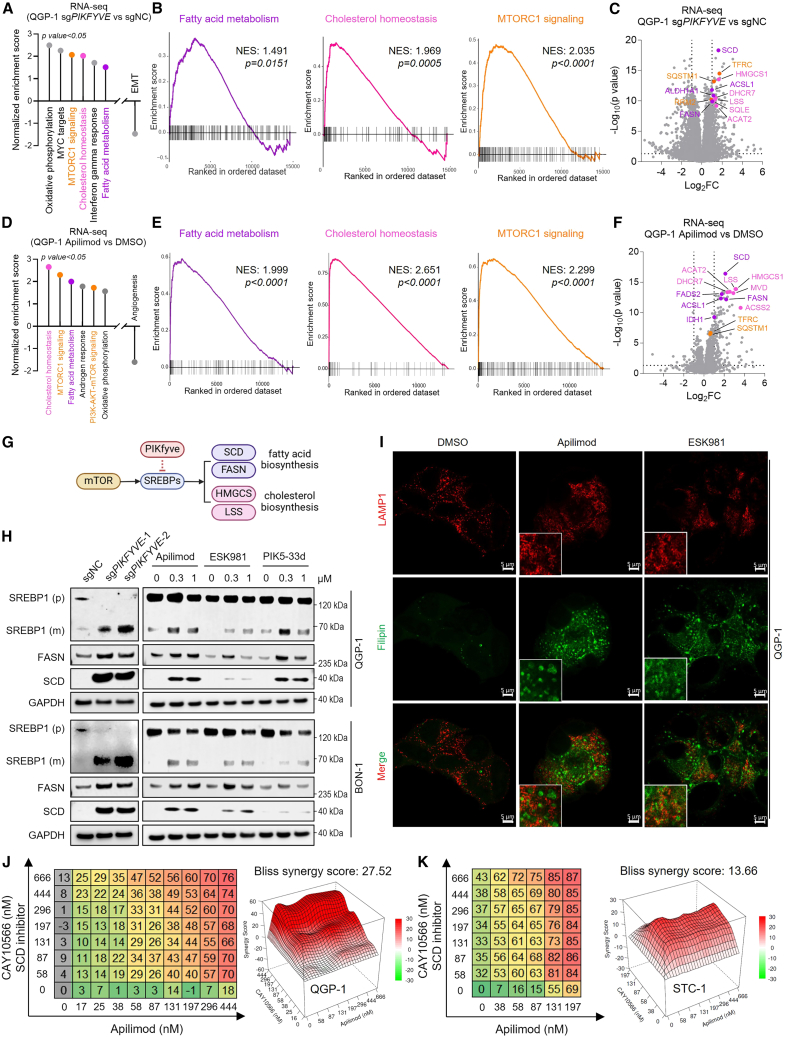
PIKfyve mediates lipid homeostasis in GEP-NETs (A) Pathway enrichment analysis of RNA-seq from QGP-1 cells following CRISPRi-mediated *PIKFYVE* knockdown. (B) GSEA of fatty acid metabolism, cholesterol homeostasis, and mTORC1 signaling after CRISPRi-mediated *PIKFYVE* knockdown in QGP-1 cells. (C) Volcano plot of differentially expressed genes highlighting fatty acid metabolism (violet), cholesterol homeostasis (rose), or mTORC1 signaling pathways (orange). (D) Pathway enrichment analysis of RNA-seq from QGP-1 cells treated with PIKfyve inhibitor apilimod (1 μM, 8 h). (E) GSEA of fatty acid metabolism, cholesterol homeostasis, and mTORC1 signaling following apilimod treatment. (F) Volcano plots of differentially expressed genes from QGP-1 cells after apilimod treatment, highlighting fatty acid metabolism (violet), cholesterol homeostasis (rose), or mTORC1 signaling pathways (orange). (G) Schematic illustrating SREBP- and mTOR-dependent regulation of fatty acid cholesterol biosynthesis. (H) Immunoblot showing PIKfyve, premature SREBP1 (p), mature SREBP1 (m), FASN, and SCD expression in QGP-1 and BON-1 cells following genetic or pharmacological PIKfyve inhibition (inhibitors: apilimod, ESK981; degrader: PIK5-33d. 8-h treatment). GAPDH was used as a loading control. (I) LAMP1 and filipin (cholesterol probe) staining showing lysosomal cholesterol accumulation after apilimod or ESK981 treatment for 24 h at 1 μM. Scale bars: 5 μm. (J and K) Synergy analyses of apilimod and the SCD inhibitor (CAY10566) in QGP-1 (J) and STC-1 (K) cells, shown as dose-response heatmaps and 3D synergy plots.

PIKfyve was reported to regulate lipid homeostasis in PDAC,^21^ but its role in GEP-NETs is unknown. Here, we performed RNA sequencing (RNA-seq) in QGP-1 cells using CRISPRi-mediated *PIKFYVE* knockdown. Pathway enrichment analysis revealed enrichment of fatty acid metabolism, cholesterol homeostasis, and mTORC1 signaling upon *PIKFYVE* knockdown (Figure 3A), which was confirmed by gene set enrichment analysis (GSEA) (Figure 3B). Specifically, lipid-metabolism-related genes, such as *SCD*, *FASN*, and *HMGCS1,* were among the top upregulated genes after *PIKFYVE* knockdown (Figure 3C). This finding was validated in QGP-1 cells and other GEP-NET cell lines by qPCR analysis (Figures S2A–S2C). Importantly, these findings were recapitulated with pharmacological inhibition of PIKfyve by apilimod in QGP-1 cells using RNA-seq analysis (Figures 3D–3F). Upregulation of genes involved in lipid and cholesterol metabolism was validated in other GEP-NET cell lines following apilimod and ESK981 treatment (Figures S2D–S2F). Whole-cell proteomics confirmed increased levels of fatty acid (SQLE and SCD) and cholesterol-related proteins (HMGCS1 and LSS) after apilimod treatment, in line with transcript changes (Figure S2G). These results indicate that PIKfyve mediates lipid homeostasis in GEP-NET cells, and both genetic and pharmacological inhibition of PIKfyve induce metabolic reprogramming.

SREBPs are key transcription factors regulating fatty acid and cholesterol biosynthesis.^32^ To determine whether PIKfyve-inhibition-induced fatty acid and cholesterol biosynthesis involves SREBPs (Figure 3G), we performed immunoblot experiments from QGP-1, BON-1, and GOT-1 cells with CRISPRi-mediated *PIKFYVE* knockdown and found that SREPB1 was cleaved and activated upon PIKfyve loss; similar results were obtained with pharmacological inhibition of PIKfyve using inhibitors (apilimod, ESK981) or a degrader (PIK5-33d) (Figures 3H and S2H–S2J). Notably, cholesterol can be transported from lysosomes to peroxisomes via lysosome-peroxisome membrane contact sites.^33^ We hypothesized that lipid biosynthesis upregulation compensates for disrupted lysosomal function. To test this hypothesis, we performed immunofluorescent staining of LAMP1 (a lysosomal marker) and co-stained with a cholesterol-specific probe (filipin) to evaluate cholesterol distribution in QGP-1 and BON-1 cells following treatment with PIKfyve inhibitors (apilimod, ESK981). Upon PIKfyve inhibition, filipin staining showed cholesterol accumulation within lysosomes, as indicated by colocalization with LAMP1-positive organelles (Figures 3I and S2K), indicating impaired cholesterol trafficking and utilization.

Since lipid biosynthesis may compensate for PIKfyve inhibition, we tested whether disrupting lipid homeostasis creates a metabolic vulnerability in GEP-NETs. We concurrently inhibited PIKfyve (with apilimod or ESK981) and fatty acid metabolism (with TVB2640 inhibiting FASN or CAY10566 targeting SCD) in multiple GEP-NET cell lines (QGP-1, BON-1, GOT-1, and STC-1). Remarkably, both FASN and SCD inhibitors synergized with PIKfyve inhibitors to significantly reduce cell viability across all GEP-NET cell lines tested (Figures 3J, 3K, and S2L–S2R). Real-time proliferation assays confirmed that genetic or pharmacological inhibition of PIKfyve supported these synergistic effects (Figures S2S–S2V). These findings suggest that disrupting lipid biosynthesis pathways exacerbates the metabolic stress induced by PIKfyve inhibition, rendering GEP-NETs more vulnerable to lipid homeostasis disturbances.

### mTOR inhibition decreases SREBP1 expression and triggers ferritinophagy in GEP-NETs

**Figure 4:**
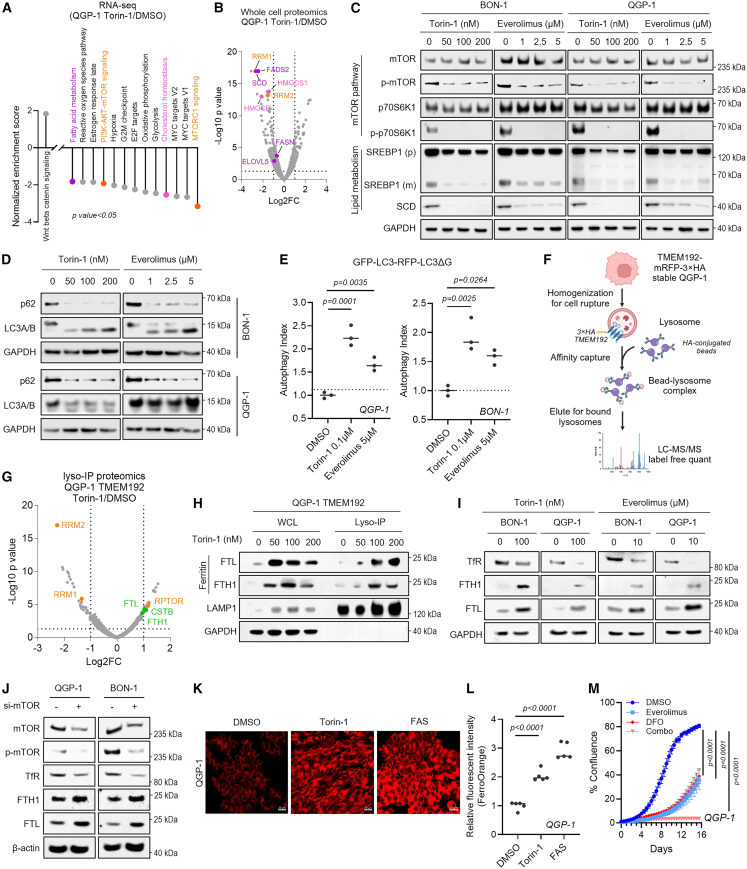
Inhibition of the mTOR pathway suppresses the SREBP1 pathway and triggers ferritinophagy (A) Pathway enrichment analysis of RNA-seq in QGP-1 cells treated with Torin-1 (0.1 μM) for 8 h (B) Volcano plot of differentially expressed proteins from whole-cell proteomics of QGP-1 cells after Torin-1 treatment (0.1 μM, 24 h) (specified in Table S3), highlighting fatty acid metabolism, cholesterol homeostasis, and mTORC1 signaling pathways. (C) Immunoblot analysis of mTOR pathway and lipid metabolism proteins in BON-1 and QGP-1 cells after 24 h of the indicated treatment. GAPDH was used as a loading control. (D) Immunoblot showing autophagy-related proteins in BON-1 and QGP-1 cells following Torin-1 or everolimus treatment for 24 h. GAPDH was used as a loading control. (E) Tandem fluorescent reporter assay assessing autophagic flux in QGP-1 and BON-1 cells treated with Torin-1 (0.1 μM) or everolimus (5 μM) for 24 h. One-way ANOVA. (F) Schematic of TMEM192-labeled cells for lysosome isolation and proteomic analysis. (G) Volcano plot of differentially expressed lysosomal proteins of QGP-1 TMEM192 cells treated with Torin-1 (0.1 μM, 24 h) (specified in Table S3), highlighting proteins in mTORC1 signaling (orange) and ferritinophagy (green). (H) Immunoblot validating increased ferritin levels (FTL and FTH1) in QGP-1 TMEM192 cells following Torin-1 treatment for 24 h. GAPDH was used as a loading control for whole-cell lysates, while LAMP1 served as a loading control for lysosomal samples. (I and J) Immunoblot analysis of ferritin and transferrin levels in BON-1 and QGP-1 cells treated with mTOR inhibitor (Torin-1, everolimus) for 24 h or siRNA-mediated knockdown of mTOR. GAPDH and β-actin were used as loading controls. (K) Representative image of FerroOrange staining in QGP-1 cells with the indicated compounds. Torin-1 was used at 100 nM, and Ferrous ammonium sulfate (FAS) was used as a positive control (50 μM). Scale bars: 20 μm. (L) Intracellular iron levels measured using FerroOrange dye via flow cytometry in QGP-1 cells treated with the indicated compounds. Torin-1 was used at 100 nM, and FAS was used at 100 μM. One-way ANOVA. (M) Confluence assay showing enhanced growth inhibition by everolimus (5 μM) combined with iron chelating agent deferoxamine (DFO, 20 μM). Data are presented as mean ± SD (*n* = 3∼4). Two-way ANOVA.

Our RNA-seq results suggest that mTOR signaling is activated upon PIKfyve inhibition in GEP-NETs, and mTOR is a known regulator of SREBPs and inhibitor of autophagy.^34^ Consequently, inhibition of mTOR activity leads to a marked disruption of lipid homeostasis while simultaneously enhancing autophagy across various cancer types, especially in pancreatic cancer.^16^^,^^35^^,^^36^ We, thus, hypothesized that mTOR inhibition would impact similar pathways in GEP-NETs. RNA-seq analysis in QGP-1 cells treated with mTOR inhibitor Torin-1 showed decreased fatty acid metabolism and cholesterol pathways, with *SCD* as the most downregulated gene. Of note, the upstream transcription factor of fatty acid and cholesterol metabolism, *SREBF1* (coding SREBP1 protein), was also downregulated by Torin-1 (Figures 4A and S3A). Key targets in fatty acid and cholesterol metabolism, including *ACACA*, *FASN*, *HMGCS1*, and *SCD*, were confirmed by qPCR to be downregulated in BON-1 and QGP-1 cells with Torin-1 and everolimus treatment (Figure S3B). Proteomics analysis on whole-cell lysates of QGP-1 cells showed that SCD and HMGCS1 protein levels were also reduced (Figure 4B). Immunoblots revealed dose-dependent reductions in total and mature SREBP1 and the downstream target SCD following Torin-1 and everolimus treatment (Figure 4C), along with activation of autophagy, as evidenced by increased lipidated LC3A/B and decreased p62 (Figures 4D and S3C). GFP-LC3-RFP-LC3ΔG^37^ reporter assays confirmed elevated autophagic flux (Figure 4E). These results collectively show that the mTOR pathway is an upstream regulator of SREBP1 and suppresses autophagy in GEP-NETs.

To further elucidate the role of the lysosome in autophagy activation upon mTOR inhibition, intact lysosomes were isolated using Lyso-IP,^38^ and proteomic analysis was performed using mass spectrometry in QGP-1 TMEM192 cells treated with Torin-1 (Figures 4F, 4G, and S3D). Lysosomal proteomics revealed significant enrichment of iron storage protein ferritin heavy chain (FTH1) and ferritin light chain (FTL) upon mTOR inhibition (Figure 4G), suggesting increased levels of bioactive ferrous iron (Fe^2+^) upon mTOR inhibition.^39^ Ferritinophagy is a form of selective autophagy in which ferritin is delivered to lysosomes in an autophagy-dependent manner for degradation, thereby regulating intracellular iron bioavailability.^40^ Immunoblots of whole-cell lysates and lysosomal fractions confirmed the elevated FTL and FTH1 levels in lysosomes following Torin-1 treatment (Figure 4H). Similarly, everolimus treatment increased FTL and FTH1 levels in lysosomal fractions (Figure S3E) and decreased transferrin receptor (TfR) levels in whole-cell lysates (Figure 4I). Genetic knockdown of *MTOR* in QGP-1, BON-1, and GOT-1 cells resulted in increased FTL and FTH1 levels and decreased TfR expression (Figures 4J and S3F). These data suggest that mTOR suppression triggers ferritinophagy in GEP-NETs, which regulates intracellular iron homeostasis.

Previous reports suggest that elevation of ferritin^41^ and reduction of TfR^42^ result in cellular iron accumulation. We postulated that mTOR-inhibition-induced cellular iron storage reflects disrupted cellular iron homeostasis. Using FerroOrange staining in live cells (Figure 4K) and quantification by flow cytometry (Figure 4L), we observed that Torin-1 significantly increased intracellular iron levels, suggesting activation of ferritinophagy. Furthermore, iron deprivation by the iron chelator deferoxamine (DFO) synergized with everolimus to suppress QGP-1 and BON-1 cell growth (Figures 4M and S3G), indicating that mTOR inhibition sustains intracellular iron to support proliferation.

Iron homeostasis is regulated by iron regulatory proteins (IRPs), particularly IRP2 (*IREB2*), which post-transcriptionally controls FTH1 and FTL expression via iron response elements (IREs).^43^ IRP2 protein can be degraded by the E3 ubiquitin ligase F box and leucine-rich repeat protein 5 (FBXL5), relieving suppression of ferritin expression.^44^ Additionally, tristetraprolin (TTP) destabilizes transferrin receptor 1 (*TFRC*) mRNA downstream of mTOR through a mechanism distinct from the classical IRP-IRE system.^41^ To define iron regulation following mTOR inhibition, we performed time course analyses of intracellular iron and iron regulatory genes. As expected, increased iron levels were observed over the course of mTOR inhibitor treatment (Figures S3H and S3I), while *IREB2* mRNA was reduced (Figure S3J). Consistent with previous work,^42^ we observed a steady decrease in *TFRC* mRNA levels following Torin-1 or everolimus treatment, whereas *SLC40A1* expression (encoding the FPN protein) was transiently upregulated before declining (Figure S3K). Consistently, IRP2 protein levels decreased upon mTOR inhibition, accompanied by increased FTH1 and FTL protein expression (Figure S3L). Furthermore, TTP knockout rescued *TFRC* mRNA expression following mTOR inhibition, indicating that mTOR regulates TfR1 through TTP (Figure S3M). These data indicate that mTOR regulates iron homeostasis in GEP-NETs through coordinated control of IRP2-ferritin and TTP-TFRC pathways.

### PIKfyve inhibition abrogates mTOR-inhibition-induced ferritinophagy

**Figure 5:**
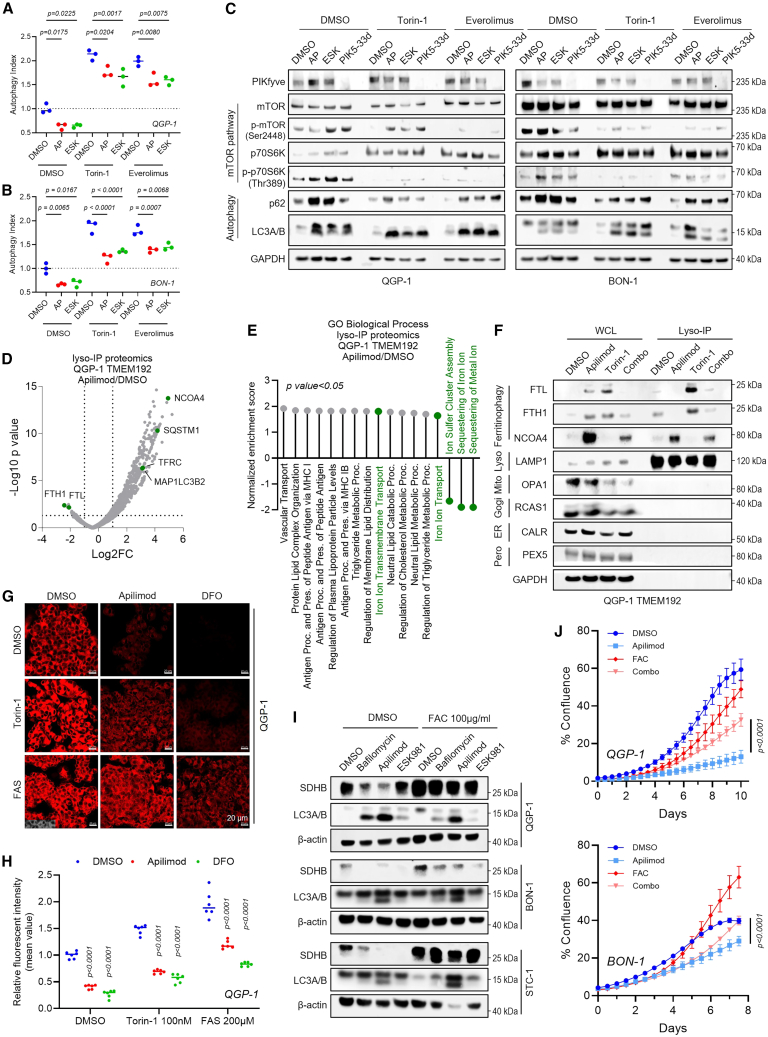
PIKfyve blockade abrogates mTOR-inhibition-induced ferritinophagy (A and B) Autophagic flux in QGP-1 (A) and BON-1 (B) cells treated with DMSO, apilimod (AP), ESK981 (ESK), or combinations with Torin-1 (0.1 μM) or everolimus (5 μM) for 24 h. Statistical analysis using one-way ANOVA. (C) Immunoblot analysis of mTOR signaling and autophagy markers in QGP-1 and BON-1 cells treated with mTOR inhibitors with or without PIKfyve antagonists. GAPDH served as a loading control. (D) Volcano plot of differentially expressed lysosomal proteins in QGP-1 TMEM192 cells treated with or without apilimod (1 μM, 24 h) (specified in Table S3). (E) Pathway enrichment analysis of lysosomal proteomics highlighting ferritinophagy-related pathways. (F) Immunoblot validation of decreased ferritin levels (FTL and FTH1) in QGP-1 TMEM192 cells following the indicated treatment for 24 h. GAPDH served as loading control for whole-cell lysates; LAMP1 served as loading control for lysosomal samples. (G and H) Intracellular iron levels in QGP-1 cells assessed by FerroOrange staining (G) and flow cytometry (H). FAS served as a positive control; deferoxamine (DFO) served as a negative control. Scale bars: 20 μm. Statistical analysis using two-way ANOVA. (I) Immunoblot analysis of SDHB following changes in bioavailable iron after lysosomal or PIKfyve inhibition with or without ammonium ferric citrate (FAC) supplementation in indicated cells. β-actin served as the loading control. (J) Confluence assay showing FAC (100 μg/mL)-mediated rescue of cell growth after apilimod treatment. All conditions were supplemented with 1 μM Ferrostatin-1 to prevent the deleterious effects of excess free iron. Data presented as mean ± SD (*n* = 3). Statistical analysis using two-way ANOVA.

To evaluate whether inhibiting PIKfyve is sufficient to block mTOR-inhibition-induced ferritinophagy in GEP-NETs, we examined the cellular response of combined PIKfyve and mTOR inhibitor treatment. Autophagic flux was markedly reduced following treatment with apilimod or ESK981 under conditions also including mTOR inhibitors Torin-1 or everolimus or siRNA-mediated *PIKFYVE* knockdown (Figures 5A, 5B, and S4A–S4C). Immunoblot analysis confirmed that co-treatment with apilimod or ESK981 mitigated the mTOR-inhibition-induced autophagic activation, as evidenced by increased lipidated LC3A/B and p62 levels in the co-treated samples compared to mTOR inhibitor alone (Figures 5C and S4D). PIKfyve inhibition also elevated p-mTOR and p-p70S6K expression, consistent with mTOR pathway activation (Figures 3B and 3E). Lysosomal proteomics in QGP-1 TMEM192 cells treated with apilimod confirmed reduced autophagic flux. Notably, the most depleted proteins were FTH1 and FTL in lysosomes, while NCOA4, a key adapter protein for ferritinophagy, was enriched following apilimod treatment (Figure 5D). GO biological process analysis further indicated increased iron transport pathways and decreased iron sequestration upon PIKfyve inhibition (Figure 5E). Thus, these results suggest that PIKfyve inhibition attenuates mTOR-inhibition-induced ferritinophagy.

Immunoblot analysis in QGP-1 cells confirmed NCOA4 enrichment in whole-cell lysates and lysosomal fractions and reduced FTL and FTH1 levels in lysosomal fractions treated with apilimod (Figure 5F). Attenuated FTL and FTH1 levels were observed with combined Torin-1 and apilimod treatment compared to Torin-1 alone (Figure 5F), suggesting that PIKfyve inhibition disrupts mTOR-inhibition-induced ferritinophagy and may alter intracellular iron concentrations. Consistently, FerroOrange staining revealed that apilimod reduced the Torin-1-induced increase in cellular iron (Figures 5G and 5H).

Activation of ferritinophagy increases the labile iron pool (LIP), and mitochondrial iron-sulfur clusters (ISCs) indicate iron homeostasis.^45^ Previous studies have shown that among the five mitochondrial electron transport chain complexes, only the ISC in complex II, specifically SDHB, is reduced following the loss of autophagy.^46^ Consistently, SDHB expression was reduced by bafilomycin (lysosome inhibitor), apilimod, or ESK981 and partially restored by ferric ammonium citrate (FAC) supplementation (Figure 5I). Importantly, exogenous iron supplementation with FAC significantly attenuated the cell growth inhibition induced by apilimod in GEP-NETs (Figure 5J). Conversely, NCOA4 knockdown modestly increased sensitivity to everolimus (Figures S4E–S4H). Together, these results highlight a critical role for autophagic lysosome-dependent iron delivery in supporting ferritinophagy and cell proliferation under mTOR pathway suppression.

### Combined mTOR and PIKfyve inhibition exerts synergistic anti-tumor effects in GEP-NETs

**Figure 6:**
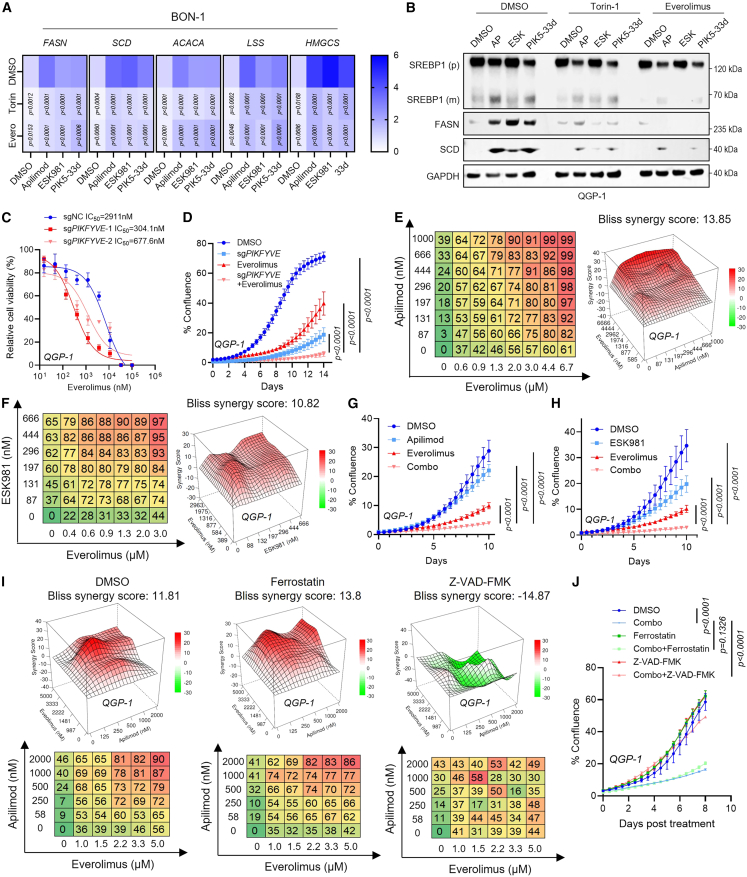
Dual inhibition of mTOR and PIKfyve triggers synthetic lethality *in vitro* (A) Heatmap showing RT-qPCR analysis of lipid metabolism targets in BON-1 cells treated with mTOR inhibitors (Torin: Torin-1; Evero: everolimus) with or without PIKfyve antagonists. Statistical analysis using two-way ANOVA. (B) Immunoblot analysis of lipid metabolism proteins in QGP-1 cells treated as (A). GAPDH, loading control. (C) Everolimus IC_50_ curves in QGP-1 cells with or without CRISPRi-mediated *PIKFYVE* knockdown. Inset shows IC_50_ values. (D) Confluence assay showing the efficacy of everolimus (5 μM) upon *PIKFYVE* knockdown in QGP-1 cells. Data shown are mean ± SD (*n* = 3). Two-way ANOVA. (E and F) 3D synergy plots and heatmaps for QGP-1 cells treated with everolimus and apilimod or ESK981. Red peaks indicate synergy, with the average synergy score shown. (G and H) Confluence assay showing synergistic effect of apilimod (1 μM) or ESK981 (250 nM) combined with everolimus (5 μM). Data presented as mean ± SD (*n* = 3). Statistical analysis using two-way ANOVA. (I) 3D synergy plots and heatmaps for apilimod and everolimus with rescue by DMSO, Ferrostatin-1 (1 μM), or Z-VAD-FMK (10 μM). (J) Confluence assay showing synergistic effect of apilimod (1 μM) and everolimus (5 μM) rescued with DMSO, Ferrostatin-1 (1 μM), or Z-VAD-FMK (10 μM). Data presented as mean ± SD (*n* = 3). Statistical analysis using two-way ANOVA.

We next investigated whether the opposing effects of PIKfyve and mTOR signaling on lipid homeostasis and lysosomal iron flux could be therapeutically leveraged. We confirmed that mTOR inhibitors (Torin-1 and everolimus) effectively suppressed lipid homeostasis genes (*FASN*, *SCD*, *ACACA*, *LSS*, and *HMGCS*) that were upregulated by PIKfyve inhibition (Figures 6A, S5A, and S5B). Protein levels of FASN, SCD, and mature SREBP1 were similarly blocked by mTOR inhibition (Figures 6B and S5C). Additionally, CRISPRi-mediated *PIKFYVE* knockdown markedly increased everolimus sensitivity, lowering the IC_50_ values from micromolar to nanomolar levels (Figures 6C and S5D), and synergistically suppressed cell proliferation in real-time assays (Figure 6D). Pharmacological co-inhibition with apilimod or ESK981 and everolimus exhibited a strong synergistic anti-proliferative effect, evidenced by Bliss synergy analysis and real-time cell proliferation assays (Figures 6E–6H and S5E–S5G). Moreover, combination treatment with the apoptosis inhibitor Z-VAD-FMK, but not the ferroptosis inhibitor Ferrostatin-1, rescued proliferation in QGP-1 and BON-1 cells, indicating that dual PIKfyve and mTOR inhibition induces apoptosis rather than ferroptosis (Figures 6I, 6J, and S5H–S5K). These results highlight a therapeutic vulnerability of GEP-NETs to combined PIKfyve and mTOR inhibition.

Clinically, mTOR inhibitors are the standard-of-care treatment for tumors reliant on mTOR signaling, here termed “mTOR-driven cancers.” We hypothesized that they may benefit from dual inhibition of mTOR and PIKfyve. We examined renal cancer carcinoma (786-O, Caki-1), fibrosarcoma (HT-1080), and liver cancer (Hep-G2) cell lines for dual mTOR and PIKfyve inhibition. Bliss synergy analysis of everolimus with apilimod or ESK981 showed strong synergism in all mTOR-driven lines (Figures S6A–S6K), whereas no synergy was observed in the non-mTOR-driven neuroendocrine prostate cancer line NCI-H660 (Figures S6L and S6M).

**Figure 7:**
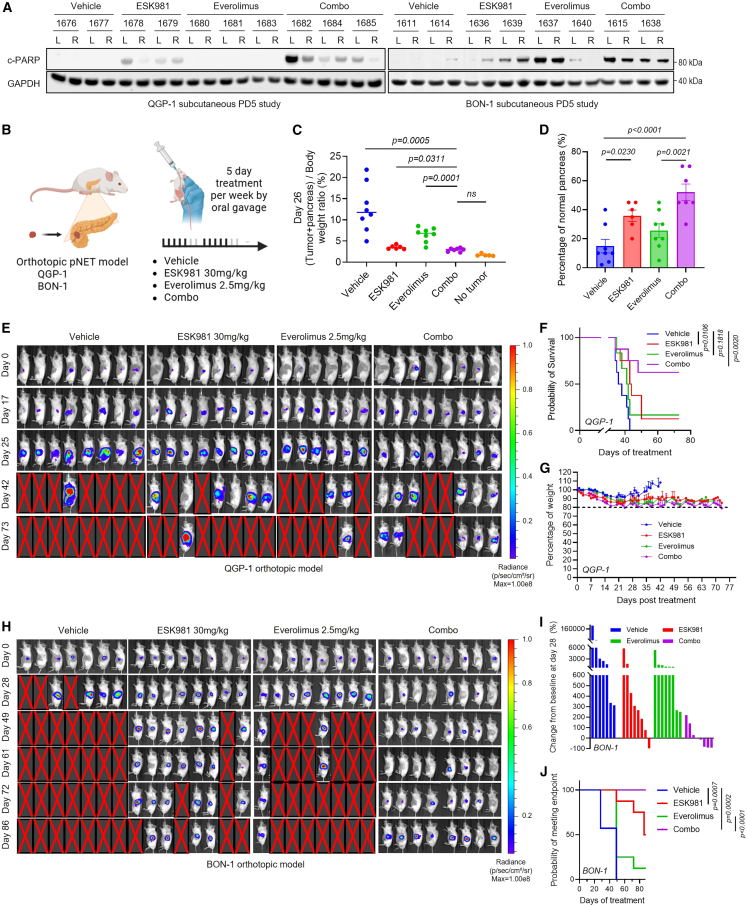
Combinatorial targeting of mTOR and PIKfyve exerts synergistic effects *in vivo* in GEP-NETs (A) Immunoblot of QGP-1 and BON-1 CDX tumors after 5 days (PD5) of treatment with vehicle, ESK981 (30 mg/kg), everolimus (2.5 mg/kg), or the combination, showing cleaved PARP (c-PARP); GAPDH, loading control. (B) Schematic of orthotopic pancreatic neuroendocrine tumor model (QGP-1 or BON-1) and treatment regimens. (C) Tumor-to-body weight ratio of pancreas from QGP-1 orthotopic model. (D) Percentage normal pancreas area from the study in (C). Data presented as mean ± SEM. *p* values calculated using one-way ANOVA. (E) Bioluminescence imaging (BLI) of QGP-1 orthotopic tumors across treatment groups. (F) Kaplan-Meier survival curves of QGP-1 tumor-bearing mice. Data expressed as means ± SEM. *p* values calculated using two-way repeated-measures ANOVA. (G) Percentage body weight changes of QGP-1 tumor-bearing mice. Data presented as mean ± SEM. (H) BLI of BON-1 orthotopic tumors. Data presented as mean ± SEM. (I) Percentage change in BLI signal on day 28 compared to day 0 for BON-1 orthotopic model shown in (H). (J) Kaplan-Meier survival curves of BON-1 tumor-bearing mice. Data expressed as means ± SEM. *p* values calculated using two-way repeated-measures ANOVA.

Building on our promising *in vitro* results, we evaluated the *in vivo* anti-tumor efficacy of combined mTOR and PIKfyve inhibition in a series of GEP-NET xenograft models. A pharmacodynamic study after 5 days of treatment with vehicle, ESK981, everolimus, or combined ESK981 and everolimus indicated that combined PIKfyve and mTOR inhibition led to PARP cleavage in both QGP-1 and BON-1 subcutaneous CDX models, indicative of enhanced apoptosis (Figure 7A). Conversely, ferroptosis markers (GPX4 and 4-HNE) were unchanged (Figure S7A), confirming that the cell death induced by co-targeting of PIKfyve and mTOR is apoptosis-dependent rather than ferroptosis. Additionally, ferritin (FTL and FTH1) accumulated in both QGP-1 and BON-1 tumors treated *in vivo* with combined ESK981 and 2.5 mg/kg everolimus, together with NCOA4 depletion, confirming ferritinophagy blockade (Figure S7B). At a higher therapeutic dose of everolimus (5 mg/kg), TfR expression was significantly downregulated following mTOR inhibition, and this effect was rescued by ESK981; SCD was also downregulated in the combinational group, indicating lipid metabolism suppression (Figure S7C). These findings reveal functional iron deficiency and impaired lipid metabolism in tumors following dual PIKfyve and mTOR inhibition.

To evaluate the anti-tumor efficacy of combined PIKfyve and mTOR inhibition, orthotopic pancreatic NET *in vivo* models were established with QGP-1 and BON-1 cells (Figure 7B). After 4 weeks of treatment in QGP-1 tumor-bearing mice, ESK981 or everolimus monotherapy reduced tumor burden; strikingly, the combination of ESK981 and everolimus further reduced tumor burden in the pancreas to near non-tumor-bearing levels (Figures 7C and S7D). Likewise, histological analysis by synaptophysin IHC and H&E staining revealed a marked increase in normal pancreatic area, with extensive necrosis in the tumor region, evidenced by cyst-like structures following the combination treatment (Figures 7D, S7E, and S7F). Importantly, combination therapy was well tolerated, with no significant body weight loss or organ toxicity (Figures S7F and S7G).

We then performed an independent survival study using the QGP-1 pancreatic orthotopic model and monitored tumor burden with bioluminescence imaging (BLI). Analysis of BLI signals showed that combined ESK981 and everolimus markedly decreased tumor burden and prolonged survival of tumor-bearing mice compared to vehicle or either monotherapy (Figures 7E and 7F) without significantly affecting host body weights (Figure 7G). Similar results were observed in the BON-1 pancreatic orthotopic model, where combination treatment reduced tumor burden at day 28, enhanced survival, and was well tolerated (Figures 7H–7J and S7H).

PIKfyve inhibition can enhance anti-tumor immunity through upregulation of CXCL10 and major histocompatibility complex (MHC) class I.^18^^,^^20^ Considering the lack of an immunocompetent *in vivo* model for GEP-NET in our study, we tested this hypothesis using *in vitro* models in QGP-1 and STC-1 cells. The results indicated that combination treatment of ESK981 and everolimus markedly increased CXCL10 and surface MHC-I expression, suggesting a potential enhancement of immune cell infiltration and improved efficacy of immune checkpoint blockade therapy (Figures S7I–S7L).

Collectively, our results show that mTOR inhibition induces a synthetic vulnerability to PIKfyve inhibition in GEP-NETs. Given the current clinical approval of everolimus and the phase I-cleared ESK981, this combination strategy holds promise for future clinical trials. Furthermore, targeting lipid metabolism and lysosomal function through this dual inhibition approach offers a promising route for therapeutic intervention to overcome resistance mechanisms frequently encountered during mTOR inhibitor monotherapy.

## Discussion

The incidence of GEP-NETs has increased nearly 4-fold over recent decades,^47^ yet survival remains largely unchanged, underscoring a pressing need for improved therapies. Using an unbiased kinome-wide CRISPR-Cas9 screen, we identify PIKfyve as a therapeutic vulnerability, with marked overexpression in GEP-NET tissues. Mechanistically, PIKfyve maintains lysosome-dependent autophagic flux and suppresses SREBP1-driven lipogenesis; its inhibition impairs autophagy and triggers compensatory fatty acid and cholesterol biosynthesis. mTOR inhibitors such as everolimus remain a cornerstone of treatment for advanced GEP-NETs,^48^ though their benefits are often blunted by acquired resistance and limited survival impact.^49^ We show that mTOR inhibition induces ferritinophagy and iron-dependent survival pathways, which are abrogated by PIKfyve inhibition. The opposing effects of PIKfyve and mTOR inhibition on lipid and iron homeostasis converge to trigger cell death, establishing a synthetic lethal interaction and a strong rationale for dual therapy with ESK981 and everolimus in GEP-NETs.

Autophagy has dual roles in cancer, suppressing tumor initiation but supporting tumor growth under high metabolic demand.^50^ mTOR directly phosphorylates the ULK^51^ and VPS34 complexes^52^^,^^53^ to suppress autophagic processes. In malignant cells, mTOR inhibitors derepress ULK and VPS34 to enable the autophagic cascade,^54^ contributing to therapy resistance and transient tumor suppression.^55^ In GEP-NETs, we find that autophagy induced by mTOR inhibitors involves ferritinophagy with NCOA4-dependent lysosomal delivery of ferritin to enhance intracellular iron levels. mTORC1-dependent roles in ferritinophagy have been previously reported in tubular epithelial cells^42^ and hematopoietic stem cells.^56^ In pNET, a previous study demonstrated that metal homeostasis modulates tumor growth, including copper.^57^ Our study on iron homeostasis further expands this concept. Ferritinophagy facilitates the degradation of ferritin within lysosomes, releasing bioavailable ferrous iron (Fe^2+^) to support mitochondrial respiration and energy metabolism, particularly as an adaptive response under mTOR inhibition.^58^^,^^59^ This study reveals that mTORC1-regulated ferritinophagy also occurs in tumor cells, with a prominent role in GEP-NETs.

Autophagy inhibition has been tested clinically using hydroxychloroquine (HCQ), which raises lysosomal pH to block autophagy. Single-agent trials largely failed because HCQ could not achieve therapeutic concentrations in patients.^50^ Nevertheless, combination strategies with mTOR inhibitors have been explored, such as everolimus and HCQ in renal cell carcinoma (NCT01510119) and recurrent breast cancer (NCT03032406). Although HCQ is a suboptimal autophagy inhibitor in patients, it enhanced the anti-tumor effect of everolimus in the phase I/II renal cell carcinoma study, and the trial met its primary endpoint of over 40% of patients with progression-free survival greater than 6 months.^60^ These findings validate the strategy of co-targeting autophagy and mTOR pathways in cancer treatment and strongly suggest that combining everolimus with a more potent autophagy inhibitor could yield stronger anti-tumor effects.

Our findings identify PIKfyve as a robust therapeutic target in the autophagy pathway. The clinical grade PIKfyve inhibitor, ESK981, is under phase II clinical investigation in multiple solid tumors (NCT05988918). PIKfyve inhibition disrupts autophagic flux and induces lipid metabolic activity, revealing a metabolic vulnerability that can be exploited through co-targeting with mTOR inhibitors. These findings suggest that dual inhibition of mTOR and PIKfyve exacerbates metabolic stress in GEP-NET cells by disrupting ferritinophagy and lipid metabolism. Extending these discoveries, our results show that everolimus synergizes with PIKfyve inhibitors in other *in vitro* models of mTOR-driven cancers, such as renal cancers and fibrosarcomas. However, whether the synergistic lethality is mechanistically dependent on SREBPs and ferritinophagy remains to be determined, as well as validation in preclinical models of these cancer types.

Collectively, our findings uncover a critical metabolic vulnerability in GEP-NETs arising from the functional interplay between PIKfyve and mTOR signaling. PIKfyve emerges as a central regulator of lipid metabolism and lysosomal homeostasis, and its inhibition disrupts survival pathways activated by mTOR blockade, converting a largely cytostatic response into cytotoxicity. These results nominate PIKfyve as both a therapeutic target and a co-target to enhance mTOR-directed therapies. ESK981, a multi-kinase inhibitor, exerts its effects by inhibiting PIKfyve in tumor cells while simultaneously blocking angiogenesis through targeting vascular endothelial growth factor receptors (VEGFRs) in host cells, likely contributing to its monotherapy efficacy. This dual action may provide an advantage in patients, given the role of neo-angiogenesis in GEP-NET aggressiveness.^61^ Overall, the robust synergy observed in this work between ESK981 and everolimus in preclinical GEP-NET models provides a strong mechanistic and translational rationale to propel this combination into clinical investigation, offering a promising strategy to overcome therapeutic resistance and improve outcomes in patients with mTOR-driven malignancies.

### Limitations of the study

Due to the rarity of GEP-NETs and the limited model availability, we were unable to include patient-derived xenograft or syngeneic systems, and our *in vivo* studies were restricted to CDX models in immunodeficient mice, preventing assessment of immune-related effects and the tumor microenvironment. In addition, while ESK981 exhibits dual activity against PIKfyve and angiogenesis, the relative contribution of each mechanism to the anti-tumor efficacy of ESK981 in combination with everolimus could not be fully determined. In PDAC, ferritinophagy is known to be elevated and to support tumor cell growth^62^; however, exogenous iron supplementation with FAC does not reduce the proliferation inhibition induced by PIKfyve inhibitors,^21^ suggesting a context-dependent role of PIKfyve in regulating ferritinophagy. Further investigation is needed to clarify the underlying mechanisms. It also remains unclear whether overexpression of mature SREBP alone can rescue the effects of combined mTOR and PIKfyve inhibition, leaving open the possibility that additional PIKfyve- and mTOR-dependent pathways contribute to the synthetic lethal phenotype.

## Resource availability

### Lead contact

Further information and requests for resources should be directed to the lead contact, Arul M. Chinnaiyan (arul@med.umich.edu).

### Materials availability

All materials used in this paper are available from the lead contact upon request.

### Data and code availability

• CRISPR screen and RNA sequencing data generated in this study are deposited in the Gene Expression Omnibus (GEO) database with accession numbers GEO: GSE293842 and GSE293843, respectively.
• No custom code was developed in this study.
• Any additional information required to reanalyze the data reported in this paper is available from the lead contact upon request.

## Acknowledgments

We gratefully acknowledge Lanbo Xiao, Jacinda Liu, Eleanor Young, Brian Magnuson, Yi Bao, Yihan Liu, Sydney Peters, Jasmine Wisniewski, Lisa McMurry, Fengyun Su, Rui Wang, Amanda Miller, Christine Caldwell-Smith, Xia Jiang, Yunhui Cheng, Shuqin Li, and Jean Tien from the Michigan Center for Translational Pathology at the 10.13039/100007270University of Michigan for providing technical assistance. This work was supported by the following mechanisms: 10.13039/100000054National Cancer Institute (NCI) Outstanding Investigator Award R35-CA231996 (A.M.C.), 10.13039/100000005Department of Defense Idea Development Award HT9425-23-1-0084 (Y.Q.), and the Neuroendocrine Tumor Research Foundation Investigator Award (Y.Q.). C.A.L. was supported by the 10.13039/100000054NCI (R37-CA237421, R01-CA248160, and R01-CA244931). C.C. was supported by an 10.13039/100000054NCI F30 fellowship (F30CA288093) and 10.13039/100000002NIH T32 training grants (CMB: 5T32-GM145470, MSTP: T32GM00786). A.M.C. is a 10.13039/100000011Howard Hughes Medical Institute Investigator, A. Alfred Taubman Scholar, and American Cancer Society Professor.

## Author contributions

Y.Q., A.M.C., and Y.C. designed and conceived the study; Y.C. and Y.Q. performed all *in vitro* and functional genomic experiments with assistance from C.C., Y.Y., Y. Zheng, S.N.Y., F.Y., S.V., Y. Zhao, and R. Pakkan; Y.C. and Y.Y. performed all animal efficacy studies with help from Y.Q., Y. Zheng, and Y. Zhao; Y.C. and Y.Q. carried out all bioinformatic analyses with assistance from R.B. and A.C.; S.M. and Y.C. carried out the immunofluorescent staining; R.M. and R.P. carried out all histopathological evaluations and quantified all histology-based data and immunohistochemistry; X.C. generated next-generation sequencing libraries and performed the sequencing; C.L. and K.D. generated PIK5-33d compound; V.S. and C.A.L. assisted with manuscript organization; Y.Q. and A.M.C. provided resources and funding; Y.C., Y.Q., S.J.M., and A.M.C. wrote the manuscript and organized the final figures. All authors read, commented, and participated in manuscript review and editing.

## Declaration of interests

A.M.C. is a co-founder and serves on the Scientific Advisory Board (SAB) of Esanik Therapeutics, Inc. which owns proprietary rights to the clinical development of ESK981. Esanik Therapeutics, Inc. did not fund or approve the conduct of this study. A.M.C. is a co-founder and serves on the SAB of Medsyn Bio, Lynx Dx, NuLynx Therapeutics, and Flamingo Therapeutics. A.M.C. serves as an advisor to Tempus, Proteovant, Aurigene Oncology, and Ascentage Pharmaceuticals. A.M.C., Y.Q., C.A.L., C.C., K.D., and C.L. are listed as inventors on the following patents pertaining to development of methodologies and compounds targeting PIKfyve in diseases: PCT: PCT/US2021/057022 (A.M.C. and Y.Q.); PCT: PCT/US2024/017088 (A.M.C. and Y.Q.); PCT: PCT/CN2024/087809 (A.M.C., Y.Q., K.D., and C.L.), US Patent No: 63/537,996 (A.M.C. and Y.Q.), US Patent No: 63/841,641 (A.M.C., Y.Q., and Y.C.), US Patent No: PCT/CN2024/078381 (C.A.L., A.M.C., K.D., Y.Q., C.L., and C.C.).

## STAR★Methods

### Key resources table

**Table:** 

REAGENT or RESOURCE	SOURCE	IDENTIFIER
**Antibodies**		

Human PIKFyve Antibody	R&D Systems	Cat# AF7885; RRID: AB_3644519
PIKFYVE Polyclonal Antibody	Thermo Fisher Scientific	Cat# PA5-13977; RRID: AB_10986228
SQSTM1/p62 Rabbit mAb	Abclonal	Cat# A19700; RRID: AB_2862742
LC3A/B (D3U4C) Rabbit mAb	Cell Signaling Technology	Cat# 12741S; RRID: AB_2617131
LAMP1 (D2D11) Rabbit mAb	Cell Signaling Technology	Cat# 9091S; RRID: AB_2687579
LAMP1 (C54H11) Rabbit mAb	Cell Signaling Technology	Cat# 3243S; RRID: AB_2134478
Anti-Fatty Acid Synthase antibody	Abcam	Cat# ab22759; RRID: AB_732316
Anti-SREBP1 antibody	Abcam	Cat# ab28481; RRID: AB_778069
Anti-SCD1 antibody	Abcam	Cat# ab19862; RRID: AB_445179
Vinculin (E1E9V) Rabbit mAb (HRP Conjugate)	Cell Signaling Technology	Cat# 18799; RRID: AB_2714181
mTOR Antibody	Cell Signaling Technology	Cat# 2972; RRID: AB_330978
Phospho-mTOR (Ser2481) Antibody	Cell Signaling Technology	Cat# 2974; RRID: AB_2262884
p70 S6 Kinase (E8K6T) Rabbit mAb	Cell Signaling Technology	Cat# 34475; RRID: AB_2943679
Phospho-p70 S6 Kinase (Thr389) (108D2) Rabbit mAb	Cell Signaling Technology	Cat# 9234; RRID: AB_2269803
GAPDH (14C10) Rabbit mAb (HRP Conjugate)	Cell Signaling Technology	Cat# 3683; RRID:AB_1642205
Anti-Ferritin Light Chain antibody	Abcam	Cat# ab69090; RRID: AB_1523609
FTH1 Antibody	Cell Signaling Technology	Cat# 3998, RRID: AB_1903974
Transferrin Receptor/CD71 (D7G9X) Rabbit mAb	Cell Signaling Technology	Cat# 13113; RRID: AB_2715594
β-Actin (13E5) Rabbit mAb (HRP Conjugate)	Cell Signaling Technology	Cat# 5125; RRID: AB_1903890
NPC1 (E7S4N) Rabbit mAb	Cell Signaling Technology	Cat# 33422; RRID: AB_3697652
OPA1 (D6U6N) Rabbit mAb	Cell Signaling Technology	Cat# 80471; RRID: AB_2734117
Calreticulin (D3E6) Rabbit mAb	Cell Signaling Technology	Cat# 12238; RRID: AB_2688013
RCAS1 (D2B6N) Rabbit mAb	Cell Signaling Technology	Cat# 12290; RRID: AB_2736985
PEX5 (D7V4D) Rabbit mAb	Cell Signaling Technology	Cat# 83020; RRID: AB_2800006
Anti-SDHB antibody	Abcam	Cat# ab14714; RRID: AB_301432
Cleaved PARP (Asp214) (D6X6X) Rabbit mAb	Cell Signaling Technology	Cat# 94885; RRID: AB_2800237
Tristetraprolin (D1I3T) Rabbit mAb	Cell Signaling Technology	Cat# 71632; RRID: AB_2799806
NCOA4 Polyclonal Antibody	Thermo Fisher Scientific	Cat# PA5-96398; RRID: AB_2808200
NCOA4 Monoclonal Antibody	Thermo Fisher Scientific	Cat# MA5-56424; RRID: AB_3679479
GPX4 Recombinant Rabbit Monoclonal Antibody	Fisher Scientific	Cat# MA5-32827; RRID: AB_2810103
4-Hydroxynonenal Antibody	R&D Systems	Cat# MAB3249; RRID: AB_664165
Synaptophysin (D8F6H) Rabbit mAb	Cell Signaling Technology	Cat# 36406; RRID: AB_2799098
Synaptophysin Antibody	Ventana	Cat# 760–4595; RRID: AB_2857955
PE anti-human HLA-A,B,C Antibody	BioLegend	Cat# 311406; RRID: AB_314875
PE Mouse Anti-Mouse H-2K[d]	BD Biosciences	Cat# 562004; RRID: AB_10896488
PE Mouse Anti-Mouse H-2D[d]	BD Biosciences	Cat# 553580; RRID: AB_394938
Irp2 (D6E6W) Rabbit mAb	Cell Signaling Technology	Cat# 37135S; RRID: AB_2799110

**Biological samples**		

Tissue microarray (TMA)	TissueArray.com LLC	Cat# NE842 and NE921

**Chemicals, peptides, and recombinant proteins**		

Filipin complex	Sigma-Aldrich	Cat# F9765
FAS	Sigma-Aldrich	Cat# 215406
FAC	Sigma-Aldrich	Cat# F5879
Deferoxamine (DFO)	Sigma-Aldrich	Cat# D9533
SAR405	Selleck Chemicals	Cat# S7682
Everolimus	Selleck Chemicals	Cat# S1120
Torin-1	Selleck Chemicals	Cat# S2827
TVB-2640	Selleck Chemicals	Cat# S9714
Bafilomycin A1	Selleck Chemicals	Cat# S1413
Z-VAD-FMK	Selleck Chemicals	Cat# S7023
CAY10566	MedChemExpress	Cat# HY-15823
Apilimod	MedChemExpress	Cat# HY-14644
U18666A	MedChemExpress	Cat# HY-107433
Ferrostatin-1	MedChemExpress	Cat# HY-100579
D-luciferin Potassium salt	Regis Technologies Inc	Cat# 1-360222-200
Anti-HA Magnetic Beads	Thermo Fisher Scientific	Cat# 88837
FerroOrange	Dojindo	Cat# F374-12
Zombie NIR™ Fixable Viability Kit	BioLegend	Cat# 423106
Lipofectamine™ RNAiMAX Transfection Reagent	Thermo Scientific	Cat# 13778075

**Critical commercial assays**		

CellTiter-Glo® Luminescent Cell Viability Assay	Promega	Cat# G7572
TMT 10-plex Isobaric Label Reagents	Thermo Scientific	Cat# 90110
RNeasy Kits for RNA Purification	Qiagen	Cat# 74104
SuperScript™ III One-Step RT-PCR System with Platinum™ Taq DNA Polymerase	Thermo Scientific	Cat# 12574026
Fast SYBR Green Master Mix	Thermo Scientific	Cat# 4385612

**Deposited data**		

CRISPR screening data	This paper	GEO: GSE293842
RNA sequencing data	This paper	GEO: GSE293843

**Experimental models: Cell lines**		

STC-1	ATCC	RRID: CVCL_J405
HPNE	ATCC	RRID: CVCL_C466
Caki-1	ATCC	RRID: CVCL_0234
786-O	ATCC	RRID: CVCL_105
HT-1080	ATCC	RRID: CVCL_0317
Hep G2	ATCC	RRID: CVCL_0027
NCI-H660	ATCC	RRID: CVCL_1576
BON-1	Creative Biolabs	RRID: CVCL_3985
QGP-1	XenoTech	RRID: CVCL_3143
GOT-1	Yvonne Arvidsson and Ola Nilsson at the University of Gothenburg	RRID: CVCL_L306

**Experimental models: Organisms/strains**		

Mouse: CB17 SCID	Charles River Laboratories	Stock number 236

**Oligonucleotides**		

*PIKFYVE*_F: CTGAGTGATGCTGTGTGGTCAAC	Cheng et al.^21^	N/A
*PIKFYVE*_R: CAAGGACTGACACAGGCACTAG	Cheng et al.^21^	N/A
*CXCL10*_F: GGTGAGAAGAGATGTCTGAATCC	Qiao et al.^18^	N/A
*CXCL10*_R: GTCCATCCTTGGAAGCACTGCA	Qiao et al.^18^	N/A
*Cxcl10*_F: CGTCATTTTCTGCCTCATCC	Bao et al.^20^	N/A
*Cxcl10*_R: CCTATGGCCCTCATTCTCAC	Bao et al.^20^	N/A
Primers for qPCR, see Table S3	This paper	N/A
ON-TARGETplus Human siRNA SMARTPool, see Table S4	HorizonDiscovery	N/A

**Recombinant DNA**		

pMRX-IP-GFP-LC3-RFP-LC3ΔG	Addgene	Cat# 84572
LentiCRISPRv2	Addgene	Cat# 52961
Human Kinome CRISPR knockout Library	Addgene	Cat# 75314, Cat# 75315
pLV hU6-sgRNA hUbC-dCas9-KRAB-T2a-Puro	Addgene	Cat# 71236
pLJC5-Tmem192-3xHA	Addgene	Cat# 102930

**Software and algorithms**		

ImageJ	NIH	https://imagej.nih.gov/ij/
ImageStudio Lite	Li-Cor	Ver5.2
FlowJo	FlowJo Software	Version 10.8.2
SynergyFinder	Oxford Academic	https://synergyfinder.fimm.fi/synergy
PRISM	GraphPad Software	Version 10

**Deposited data**		

CRISPR screen sequencing data	This paper	GSE293842
RNA sequencing data	This paper	GSE293843

### Experimental model and study participant details

#### Cell lines

STC-1, HPNE, Caki-1, 786-O, HT-1080, Hep G2, and NCI-H660 cells were purchased from American Type Culture Collection (ATCC). BON-1 was purchased from Creative Biolabs. QGP-1 was purchased from XenoTech. GOT-1 cells were generous gifts from Yvonne Arvidsson and Ola Nilsson at the University of Gothenburg. BON-1 cells were cultured in DMEM/F12; STC-1, HT-1080, and Hep G2 were cultured in DMEM; 786-O, QGP-1, and GOT-1 were cultured in ATCC-formulated RPMI-1640; Caki-1 was cultured in McCoy’s 5A. All media were supplemented with 10% fetal bovine serum (FBS) (Hyclone, Cytavia) and 1% penicillin/streptomycin (Gibco). GOT-1 cells were also supplemented with insulin, transferrin, and selenium. NCI-H660 was cultured in ATCC RPMI supplemented with 5% FBS, 0.005 mg/mL insulin, 0.01 mg/mL transferrin, 30 nM sodium selenite, 10 nM hydrocortisone, 10 nM beta-estradiol, and extra 2 mM L-glutamine. All cell lines were incubated at 37°C with 5% CO_2_ and tested negative for mycoplasma. Human cell lines were authenticated by genotyping.

#### *In vivo* experiments

All *in vivo* studies were approved by the University of Michigan Institutional Animal Care and Use Committee (IACUC). Mice were housed in pathogen-free conditions and maintained in 12-h light/12-h dark cycles. ESK981 and everolimus were administrated by oral gavage in a concentration of 30 mg/kg and 2.5 mg/kg, respectively, following a previously described protocol.^18^ ORA-PLUS was used as vehicle treatment. ESK981, everolimus, or ORA-PLUS were given once daily at 5 days/week. ESK981 and everolimus were suspended in ORA-PLUS (Perrigo) and homogenized by sonication. Single use aliquots were frozen at −20°C to prevent freeze-thaw cycles.

For subcutaneous xenograft models, 1 million QGP-1 or BON-1 cells were resuspended in 100 μL serum free medium with 50% Matrigel. Subcutaneous tumors were generated by injection of cells into both flanks of 6–8 weeks old CB17 SCID male mice. Mice were randomized into treatment groups when average tumor volume reached 100 mm^3^. For efficacy studies, tumor volume was measured by digital caliper at least twice per week. The BON-1 CDX model was monitored for 4 weeks, and the QGP-1 CDX model was monitored for 5 weeks post treatment. The tumor volume was calculated from caliper measurements using the formula π/6 (width^2^ × length). For pharmacodynamic assessment, a small cohort of tumors were collected after 5 days of treatment (PD5).

For orthotopic xenograft models, 0.5 × 10^6^ luciferase-expressing BON-1 and QGP-1 cells were suspended in 50 μL serum free medium containing 50% Matrigel (Corning 356234). Pancreatic tumors were established by directly injecting tumor cells into the tail of the pancreas of 6–8 weeks old CB17 SCID male mice. 7 days after injection, bioluminescence imaging (BLI) was measured to assess tumor burden using the IVIS Bioluminescence Imaging after intraperitoneally injecting 100 μL of 15 mg/mL D-luciferin. Mice were then randomized into treatment groups according to tumor burden. During treatment, mice were imaged for BLI signals every 2 to 4 weeks until they reached the humane endpoint (natural death for QGP-1 or BLI signal reaching 2 × 10^8^ total flux [p/s] for BON-1).

### Method details

#### Compounds

Torin-1, everolimus, apilimod, bafilomycin A1, TVB-2640, and SAR405 were purchased from Selleck Chemicals. FAC, FAS, and DFO were purchased from Sigma-Aldrich. CAY-100566 was bought from MedChemExpress. ESK981 was provided by Esanik Therapeutics. Compound details are listed in Table S4.

#### Human kinome CRISPR knockout library preparation and analysis

The human kinome CRISPR knockout libraries were purchased from Addgene (Cat#75314, Cat#75315) and amplified according to Addgene’s protocol. Lentivirus particles were generated by the University of Michigan Vector Core. To achieve 1000x coverage per guide RNA, 10 million BON-1 cells were seeded in T150 flasks with 15 mL DMEM/F12, 8 μg/mL polybrene, and the CRISPR screen library virus at MOI = 0.3 for each library. After 24 h infection, culture media was replaced with 15 mL DMEM/F12 containing 2.5 μg/mL puromycin. After 5 days of puromycin selection, 5 million cells were collected and labeled as day 0 sample for initial population. Another 5 million cells were seeded into T150 flasks and collected after 14 days. Both day 0 and day 14 samples were harvested for genomic DNA (gDNA) isolation using DNAeasy blood and tissue kit (Qiagen) in accordance with the manufacturer’s protocol.

Following gDNA isolation, Herculase II Fusion DNA Polymerase (Agilent Technologies) was used to amplify the sgRNA from 5 μg of gDNA from each sample. Two rounds of PCR were used: First-round forward primer: TTTGCATATACGATACAAGGCTG; First-round reverse primer: TCAAGATCTAGTTACGCCAAGC; Second-round forward primer: TTTCTTGGGTAGTTTGCAGTTTT; Second-round reverse primer: TCAAGATCTAGTTACGCCAAGC. The amplified DNA was then purified by Select-a-Size DNA Clean & Concentrator kit (Zymo Research) and further gel-purified by running the purified products on a 6% Novex TBE gel (Thermo) followed by isolating the DNA using Gel Breaker Tubes and Gel Filters (BioChain). The purified DNA then underwent end-repair, A-tail addition, and New England Biolabs (NEB) adapter ligation for library establishment. Finally, 2 × KAPA HiFi HotStart mix and NEB dual code barcode were used to enrich adapter-ligated DNA fragments for final library preparation, which was sent to an Illumina NovaSeq 6000 machine for sequencing.

For data analysis, putative essential genes were identified according to the ranking by comparing sgRNA abundance on day 14 with that on day 0. The sgRNAs with fewer than 100 reads were removed. Common essential genes were acquired from DepMap Portal to nominate BON-1-specific targets.

#### siRNA transfection

SMARTpool ON-TARGETplus human siRNAs targeting *PIKFYVE*, *PIK3C3*, *PTK2*, *ILK,* and *MTOR* were purchased from Horizon Discovery. Cells were seeded in a 6-well plate at 80% confluency overnight before being transfected with 25 nM of siRNA or non-targeting control using the Lipofectamine RNAiMAX reagents (Thermo Fisher). Forty-eight hours post transfection, cells were collected for further experimentation. siRNA details are listed in Table S4.

#### Incucyte proliferation assay

Single cells were seeded on 96-well (1000 cells/well) or 24-well (5000 cells/well) plates overnight before treatment. Confluence rate was monitored by the Incucyte S3 (Sartorius). The scanned images were collected under 10x magnification every 4 h to assess the cell proliferation by percent confluence change.

#### Crystal violet staining

Cells were seeded in 96-well plates and treated for 14 days as indicated. Apilimod was refreshed every 5 days due to its short half-life. At the endpoint, plates were removed from the incubator, and each well was washed with PBS. Cells were then fixed using 10% Neutral Buffered Formalin for 30 min and stained using 1% crystal violet solution staining. Finally, the wells were rinsed with a gentle stream of cold distilled water and scanned for presentation.

#### Generation of CRISPRi-mediated knockdown cell lines

CRISPRi-mediated knockdown cell lines were generated using sgRNA sequences as follows. The human sgRNA sequences are: GCTGCATGGGGCGCGAATCA for sgNC, GGCCGGTATGGGGAGCTCCA for sg*PIKFYVE*-1, and GGGAAGTCGGCCCCCGAGAG for sg*PIKFYVE*-2; these sequences were previously described and validated^21^; AGGCGATCCGAGGAGACCTT for sg*NCOA4*-1, and TTGGGCCGTAGGTTAGTGTG for sg*NCOA4*-2. The mouse sgRNA sequences are: GCTGCATGGGGCGCGAATCA for sgNC, AGAGGCTTACGCGGTAACTG for sg*Pikfyve*-1, CAGTTACCGCGTAAGCCTCT for sg*Pikfyve*-2, CCACGGCTAAGTGTCTGGGT for sg*Ncoa4*-1, and CCAGCCGGTAAGGACGAGAG for sg*Ncoa4*-2. These sgRNAs were designed using CRISPick.^63^ The sgRNAs were cloned into the pLV hU6-sgRNA hUbC-dCas9-KRAB-T2a-Puro backbone (Addgene Cat #71236) and expanded in One Shot Stbl3 chemically competent *E. coli* (ThermoFisher Scientific). sgRNA containing plasmids were verified by Sanger sequencing and packaged into lentiviruses by the University of Michigan Vector Core. BON-1, QGP-1, GOT-1, or STC-1 cells were seeded and infected with polybrene (10 μg/mL). After 24 h infection, cells were selected with puromycin (2 μg/mL for BON-1 and QGP-1, 1 μg/mL for GOT-1 and STC-1).

#### Generation of CRISPR-mediated knockout cell lines

CRISPR-mediated knockout cell lines were generated using sgRNA sequences as follows. The human sgRNA sequences are: GCTGCATGGGGCGCGAATCA for sgNC, AAGTGGCAGCGAGAGCCGTA for sg*ZFP36*-1, and GCGCAGCTCGCCCAGGCCAT for sg*ZFP36*-2. These sgRNAs were designed using CRISPick.^63^ *ZFP36* is the coding gene for Tristetraprolin (TTP). The sgRNAs were cloned into the lentiCRISPR v2 backbone (Addgene Cat #52961) and expanded in One Shot Stbl3 chemically competent *E. coli* (ThermoFisher Scientific). sgRNA containing plasmids were verified by Sanger sequencing. *ZFP36* CRISPR plasmids were used for transient transfection using Lipofectamine 3000 Reagent. BON-1 and QGP-1 cells were seeded and infected with polybrene (10 μg/mL). After 24 h infection, cells were selected with puromycin (2 μg/mL for BON-1 and QGP-1).

#### CellTiter Glo cell viability assay and IC_50_ calculation

Single cell suspensions were seeded at a density of 2000 cells/well (GOT-1) or 1000 cells/well (all other cell lines used) in 96-well plates overnight prior to drug treatment. Drugs were added at the indicated concentrations and incubated for 7 days. Cell viability was determined by CellTiter-Glo Luminescent Cell Viability Assay (Promega) according to the manufacturer’s instructions. IC_50_ values were calculated using GraphPad Prism 10.2. At least three replicates were used for each condition.

#### Synergy assays

Cells were seeded in 96-well plates and treated with the indicated combinations for 7 days prior to CellTiter Glo measurement. Four replicates were used for each condition. Each replicate was normalized to the DMSO control, and the average percent viability for each condition was calculated. The resulting values were then imported to the SynergyFinder+ web application (https://synergyfinder.org)^64^ to determine the Bliss synergy score. Average Bliss synergy scores above 10 were considered as synergy.

#### Immunohistochemistry (IHC) staining

PIKfyve IHC was performed using the Ventana DISCOVERY ULTRA system. Briefly, IHC was performed on the Ventana Discovery automated platform using formalin-fixed paraffin-embedded (FFPE) tissue sections cut at 5 μm and baked at 60°C. Afterward, slides were deparaffinized on the instrument using Discovery Wash solution (Catalog No. 950-510, Ventana Medical System, Roche Diagnostics, Indianapolis, IN, USA) at 75°C, followed by heat-induced epitope retrieval with reconstituted Discovery CC1 (Catalog No. 06414575001, Ventana Medical System, Roche Diagnostics, Indianapolis, IN, USA) at 95°C for 64 min. Endogenous peroxidase activity was blocked using Discovery Inhibitor CM (Catalog No. 760–4840, Ventana Medical System, Roche Diagnostics, Indianapolis, IN, USA) for 12 min at 37°C. A rabbit primary polyclonal PIKFVYE antibody (Catalog No. 13361-1-AP, Proteintech, Rosemont, IL, USA), diluted in Discovery Antibody Diluent (Catalog No. 760-108, Ventana Medical System, Roche Diagnostics, Indianapolis, IN, USA), was applied and incubated for 60 min at 37°C. Detection was performed using the Discovery OmniMap Anti-Rabbit HRP-RUO system (Catalog No. 760–4311, Ventana Medical System, Roche Diagnostics, Indianapolis, IN, USA) followed by signal development that was achieved by using Discovery ChromoMap DAB detection kit (Catalog No. 760-159, Ventana Medical System, Roche Diagnostics, Indianapolis, IN, USA). Counterstaining was carried out with Hematoxylin II (Catalog No. 790–2208, Ventana Medical System, Roche Diagnostics, Indianapolis, IN, USA) for 12 min and Bluing Reagent (Catalog No. 760–2037, Ventana Medical System, Roche Diagnostics, Indianapolis, IN, USA) for another 8 min. Slides were washed on the instrument, dehydrated manually through graded ethanol and xylene, and coverslipped with permanent mounting media.

Synaptophysin IHC was performed using a manual method. Briefly, 5 μm formalin-fixed tissue sections were deparaffinized and rehydrated before antigen retrieval using citrate buffer (pH 6.0). After blocking with 3% hydrogen peroxide and 10% goat serum, the slides were incubated with synaptophysin antibody overnight at 4°C. The following day, the slides were incubated with goat anti-rabbit HRP polymer, and the chromogenic signal was visualized using DAB solution. Finally, the slides were dehydrated and mounted with EcoMount (Thermo Fisher, EM897L).

#### Tissue microarray (TMA) staining and histopathological score (H-score) evaluation

Two GEP-NET TMAs (NE842 and NE921) were purchased from TissueArray.com LLC. Both TMAs were stained using the antibody and reagents as described above. Two pathologists (R. M. and R. P.) performed all morphological evaluation in this paper first independently in a blinded fashion followed by a consensus meeting to finalize the scoring. Only those spots described as normal tissue, neuroendocrine tumor, or adenocarcinoma in gastroenteropancreatic system were selected. Appropriate staining in a spot was considered when the accompanying stromal and immune cells also showed some immunopositivity. A tissue spot showing absence of staining in both tumor and accompanying stromal and immune cell was considered indeterminate for scoring and taken out of analysis.

For each finalized and selected spot, a semi-quantitative product score for the target in question was provided. The score calculated out of 300 was derived by multiplying the percentage of positive tumor cells (PP) for each staining intensity, no staining (0), weak (1+), moderate (2+), or strong (3+), and adding the values in each tumor using the formula H-score = (1×% weakly stained cells) + (2∗% ×% moderately stained cells) + (3∗% ×% strongly stained cells).

#### Western blot

Protein was collected from homogenized cells or tissue with RIPA buffer (ThermoFisher Scientific) supplemented with protease and phosphatase inhibitors (ThermoFisher Scientific). Whole-cell lysate was collected after sonication and centrifugation. Protein concentration was quantified, and 18 μg of protein was separated on SDS-PAGE system with either NuPAGE Tris-Acetate (3–8%) or Bis-Tris (4–12%) gels (ThermoFisher Scientific), followed by transfer onto polyvinylidene fluoride (PVDF) membranes. The membranes were then blocked with 5% milk in Tris-buffered saline (TBS) containing 0.1% Tween 20 (TBST) and incubated with primary antibody overnight at 4°C. The next day, the membranes were washed with TBST and incubated with HRP-conjugated secondary antibody. The membranes were imaged for results in an Odyssey FC Imaging System (LICOR bio) using ECL Select Western Blotting Detection Reagent (Cytiva). Details about the antibodies used are listed in Table S4.

#### RNA isolation and quantitative real-time PCR (qPCR)

Total RNA was extracted from cells using miRNeasy Kit (QIAGEN) following the manufacturer’s instructions. RNA concentration was determined by NanoDrop. For cDNA synthesis, 1000 ng of total RNA was synthesized into cDNA using the Maxima First Strand cDNA Synthesis (Thermo Fisher Scientific; #K1671). Quantitative real-time PCR (qPCR) was performed in triplicate using Fast SYBR Green Master Mix (Thermo Fisher Scientific; #4385612) and standard SYBR Green protocols. The reactions were conducted in a 384-well plate on a QuantStudio 7 Pro Real-Time PCR System (Thermo Fisher Scientific). The relative expression of target mRNAs was quantified using the 2^−ΔΔCT^ method and normalized to *ACTB* (human) as internal control. Primer sequences are provided in Table S4.

#### Lysosome purification

Lysosomes were purified as previously described.^65^ Briefly, QGP-1 cells with stably expressed TMEM192-3×HA (Addgene Cat #102930) were treated with DMSO, 1 μM apilimod, or 0.1 μM Torin-1 for 24 h. Cells were collected in cold KPBS buffer (136 mM KCl,10 mM KH_2_PO_4_, pH 7.25) and centrifuged, followed by mechanical homogenization. After centrifugation at 3000 rpm for 10 min, the supernatant containing HA-tagged lysosomes was collected and incubated with anti-HA-conjugated Dynabeads (Thermo Scientific, 88837) for 30 min at 4°C. Lysosomes were eluted from beads with 0.1% NP-40 KPBS buffer overnight at 4°C. Protein concentration was determined using the Pierce BCA Protein Assay Kit. Equal amounts of lysosomal protein were analyzed by immunoblotting or proteomics profiling.

#### Immunofluorescence and filipin staining

20,000 cells were seeded in 8-well Chamber Cell Culture Slides (Celltreat) overnight before the indicated treatment. After 24-h treatment, cells were washed with PBS three times, fixed with 3.2% paraformaldehyde for 15 min, and quenched with 125 mM glycine for 10 min. Samples were then permeabilized with 0.1% Triton X-100 for 5 min, blocked with 5% BSA for 1 h at 37°C, and incubated with LAMP1 primary antibody at 4°C overnight. The next day, samples were incubated with goat anti-rabbit secondary antibody and then stained with filipin complex (0.1 mg/mL) at room temperature for 2 h (Sigma-Aldrich, Cat. F9765). Chamber slides were mounted with coverslips, and representative pictures were taken using the LSM 900 confocal microscope (Zeiss).

#### Autophagic flux assay

Generation of the autophagic flux probe-labelled cells were established in BON-1 and QPG-1. Briefly, cells were infected with pMRX-IP-GFP-LC3-RFP-LC3ΔG (Addgene Cat #84572). Following puromycin selection, single cell clones were picked and genotyped for those without two LC3 fragments homologous recombination. For autophagic flux assay, 20,000 cells were seeded in clear bottom and black well 96-well plates (Corning, ref. 3603) overnight and then treated with the indicated compounds. After 24 h of incubation, plates were read in an Infinite M1000 Pro plate reader (Tecan) for GFP and RFP signals. To calculate the autophagy index, the RFP value was divided by the GFP value from each well and then normalized to the average RFP/GFP ratio in untreated control wells.

#### FerroOrange staining and quantification

FerroOrange dye (Dojindo, F374) was used to detect intracellular iron by live-cell imaging and quantification by flow cytometry. Briefly, 20,000 of the indicated cells were seeded in 8-well Chamber Cell Culture Slides (Celltreat) overnight prior to treatment with the indicated compounds for 8 h. Slides were then washed three times with HBSS and incubated with 1 μM FerroOrange for 30 min. For live-cell imaging, the slides were then imaged using LSM 900 confocal microscopes (Zeiss). For quantification by flow cytometry, cells were trypsinized and resuspended in 2% BSA and analyzed using a Sony SH800 Cell Sorter. 10,000 cells were counted for each sample.

#### Flow cytometry analysis

Tumor cells treated with the indicated compounds were harvested and resuspended in MACS buffer (PBS supplemented with 2% FBS and 2 mM EDTA). The Zombie NIR Fixable Viability Kit (BioLegend, #423106) was then applied to detect and circle out dead cells. After MHC-I antibody staining [anti-H-2Kd (BD Biosciences, #562004) and anti-H-2Dd (BD Biosciences, #553580) for the STC-1 cell line, and anti-HLA-A,B,C (clone w6/32; BioLegend, #311406) for the QGP-1 cell line], cells were washed twice with 1 mL MACS buffer and fixed in 2% paraformaldehyde (in PBS) for 15 min at room temperature. Surface expression of MHC-I was analyzed by flow cytometry (SONY SH800S), and data were analyzed with FlowJo V10.8.1.

### Quantification and statistical analysis

All statistical analyses and graphical representations were performed using GraphPad Prism. Comparisons between two groups were conducted using Student’s *t* test. For analyses involving multiple groups, one-way or two-way ANOVA was used, as appropriate. Data are presented as mean ± SEM or mean ± SD, as indicated. Corresponding statistical analyses and data presentation formats are provided in the figure legends.

#### RNA sequencing and analysis

Total RNA was extracted from all samples as described above, and RNA integrity was assessed using the Agilent Bioanalyzer with the Eukaryote Total RNA Nano Kit (Agilent Technologies, #5067-1511). Polyadenylated mRNA was isolated using Sera-Mag Magnetic Oligo(dT) particles (Sigma-Aldrich, GE38152103011150). First-strand cDNA synthesis was performed using reverse transcriptase and random primers, and double-stranded cDNA was generated through second-strand synthesis. End-repair, A-tailing, and adapter ligation were carried out using New England Biolabs (NEB) adapters, and libraries were amplified with KAPA HiFi HotStart mix and NEB dual barcodes. Sequencing was conducted on an Illumina NovaSeq 6000 platform.

Raw sequencing reads demultiplexing was performed using Illumina’s bcl2fastq software v2.20, and quality control was conducted using FastQC. Adapters and low-quality bases were moved out with Trimmomatic. Transcript-level quantifications were summarized to gene-level counts, which were normalized using TMM normalization (*calcNormFactors* in edgeR). Genes with low expression (mean TPM <1 across all groups) were filtered out. Differential expression analysis was conducted using DESeq2 to identify differentially expressed genes (DEGs) between PIKfyve knockdown vs. NC cell lines or apilimod vs. DMSO-treated QGP-1 cells. A cutoff of |log2 fold change| ≥ 1.5 and adjusted *p*-value <0.05 was applied for significance. GO enrichment was applied on differential expressed genes for enriched biological process. Enrichment of GO gene sets downloaded from MSigDB were examined with fgsea using genes ranked by logFC estimated from limma as input.

#### Proteomics profiling and analysis

Whole-cell lysates (75 μg in 75 μL RIPA buffer) or lysosome lysates (20 μg in 100 μL 0.1% NP-40 KPBS buffer) were submitted to the Proteomics Resource Facility at the University of Michigan for processing and mass spectrometry data acquisition as previously described.^66^ Briefly, proteins were digested with trypsin and labeled using TMT 10-plex Isobaric Label Reagents (Thermo Fisher Scientific, 90110) according to the manufacturer’s instructions. Three replicates were performed for each condition. The isotope-labeled samples were combined, fractionated into 12 fractions, and subjected to liquid chromatography coupled with tandem mass spectrometry (LC-MS/MS). Data acquisition was performed on an Orbitrap Ascend Tribrid mass spectrometer equipped with high-field asymmetric waveform ion mobility spectrometry (FAIMS) (Thermo Fisher Scientific) and a Vanquish *Neo* UHPLC system. Data were analyzed using Proteome Discoverer (v3.0; Thermo Fisher) and aligned against the SwissProt human protein database (20,359 entries; Homo sapiens [sp_canonical, TaxID = 9606], v2024-03-27). Proteins and peptides passing a ≤1% false discovery rate (FDR) were quantified using high-quality MS3 spectra (average signal-to-noise ratio of 10 and <50% isolation interference).

## Supplemental information

Document S1. Figures S1–S7

Table S1. Analyzed results from the CRISPR knockout kinome screen Related to Figure 1.

Table S2. Tissue microarray details and scores Related to Figure 2.

Table S3. Analyzed proteomics data Related to Figures 4, 5, and S2.

Table S4. Antibody information, qPCR primer sequences, and sgRNA sequences Related to STAR Methods.

Document S2. Article plus supplemental information
