# Supplementary material for: Targeting the ferritinophagy-lysosome axis as a therapeutic vulnerability in gastroenteropancreatic neuroendocrine tumors
Source: Cell Rep Med. 2026 Mar 24;7(4):102695. doi: 10.1016/j.xcrm.2026.102695 (PMC13130658; doi:10.1016/j.xcrm.2026.102695)
Supplement: Document S1. Figures S1–S7 [file mmc1.pdf]

Cell Reports Medicine, Volume *7*

Supplemental information

Targeting the ferritinophagy-lysosome axis as a therapeutic vulnerability in gastroenteropancreatic neuroendocrine tumors

Yizhi Cao, Caleb Cheng, Yitong Yin, Sarah N. Yee, Yang Zheng, Somnath Mahapatra, Radha Paturu, Andrej Coleski, Shannon VanAken, Fan Yang, Rüya Pakkan, Yi Zhao, Rupam Bhattacharyya, Stephanie J. Miner, Xuhong Cao, Rahul Mannan, Chungen Li, Vaibhav Sahai, Ke Ding, Costas A. Lyssiotis, Arul M. Chinnaiyan, and Yuanyuan Qiao

Figure S1

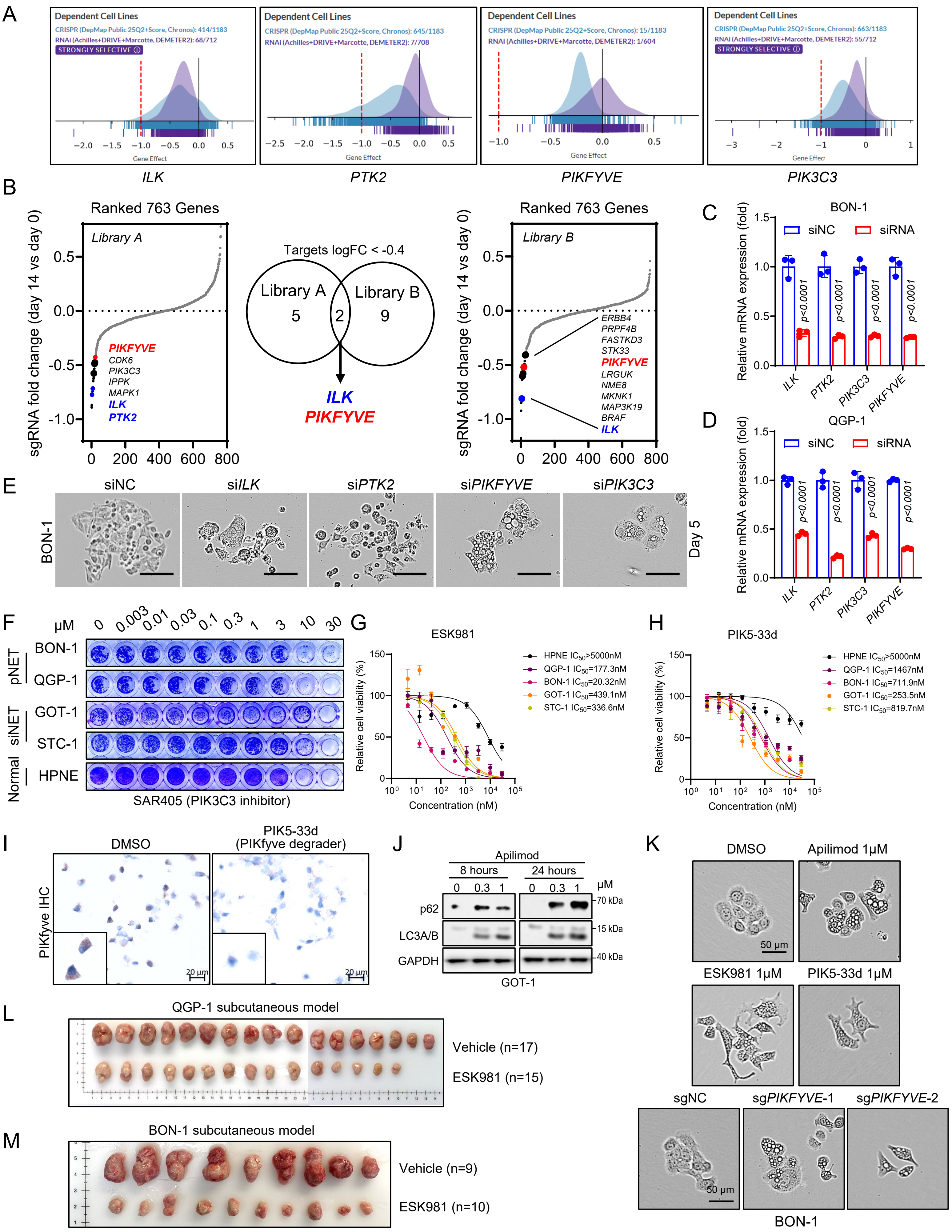

A
Dependent Cell Lines
CRISPR (DepMap Public 25Q2+Score, Chronos): 414/1183
RNAi (Achilles+DRIVE+Marcotte, DEMETER2): 68/712
STRONGLY SELECTIVE
Gene Effect
ILK
CRISPR (DepMap Public 25Q2+Score, Chronos): 645/1183
RNAi (Achilles+DRIVE+Marcotte, DEMETER2): 7/708
PTK2
CRISPR (DepMap Public 25Q2+Score, Chronos): 15/1183
RNAi (Achilles+DRIVE+Marcotte, DEMETER2): 1/604
PIKFYVE
CRISPR (DepMap Public 25Q2+Score, Chronos): 663/1183
RNAi (Achilles+DRIVE+Marcotte, DEMETER2): 55/712
PIK3C3
B
Ranked 763 Genes
Library A
sgRNA fold change (day 14 vs day 0)
PIKFYVE
CDK6
PIK3C3
IPPK
MAPK1
ILK
PTK2
Targets logFC < -0.4
Library A 5
2
Library B 9
ILK
PIKFYVE
Library B
ERBB4
PRPF4B
FASTKD3
STK33
PIKFYVE
LRGUK
NME8
MKNK1
MAP3K19
BRAF
ILK
C
BON-1
siNC
siRNA
Relative mRNA expression (fold)
p<0.0001
ILK
PTK2
PIK3C3
PIKFYVE
D
QGP-1
E
siNC
siILK
siPTK2
siPIKFYVE
siPIK3C3
BON-1
Day 5
F
μM
0
0.003
0.01
0.03
0.1
0.3
1
3
10
30
pNET
BON-1
QGP-1
siNET
GOT-1
STC-1
Normal
HPNE
SAR405 (PIK3C3 inhibitor)
G
ESK981
Relative cell viability (%)
Concentration (nM)
HPNE IC50>5000nM
QGP-1 IC50=177.3nM
BON-1 IC50=20.32nM
GOT-1 IC50=439.1nM
STC-1 IC50=336.6nM
H
PIK5-33d
HPNE IC50>5000nM
QGP-1 IC50=1467nM
BON-1 IC50=711.9nM
GOT-1 IC50=253.5nM
STC-1 IC50=819.7nM
I
DMSO
PIK5-33d
(PIKfyve degrader)
PIKfyve IHC
20 μm
J
Apilimod
8 hours
24 hours
0
0.3
1
μM
p62
LC3A/B
GAPDH
70 kDa
15 kDa
40 kDa
GOT-1
K
DMSO
Apilimod 1μM
50 μm
ESK981 1μM
PIK5-33d 1μM
sgNC
sgPIKFYVE-1
sgPIKFYVE-2
BON-1
L
QGP-1 subcutaneous model
Vehicle (n=17)
ESK981 (n=15)
M
BON-1 subcutaneous model
Vehicle (n=9)
ESK981 (n=10)

Figure S1. Kinome-wide CRISPR knockout screen identifies PIKfyve as a druggable target in GEP-NETs. Related to Figures 1 and 2.

(A) DepMap dependency plots showing selective essentiality for top hits (*ILK*, *PTK2*, *PIKFYVE*, *PIK3C3*).

(B) Snake plot and Venn diagram showing the independent analysis for library A and library B from Figure 1A. Red genes are related to the phosphatidylinositol metabolic pathway, while blue genes are related to the mTOR pathway.

(C-D) RT-qPCR analysis of BON-1 (C) and QGP-1 (D) cells after siRNA knockdown of indicated genes or control (siNC). Data represent mean ± SD from three biological replicates. Two-way ANOVA.

(E) Representative images for BON-1 cells with siNC, si*ILK*, si*PTK2*, si*PIKFYVE,* or si*PIK3C3*. Scale bar: 100 µm.

(F) Crystal violet staining showing long-term inhibitory effects of PIK3C3 inhibitor SAR405 in indicated GEP-NET cell lines and normal HPNE cells.

(G) Dose response proliferation curves of ESK981 in indicated GEP-NET cell lines and normal HPNE cells. Data presented as mean ± SD.

(H) Dose response proliferation curves of PIK5-33d in indicated GEP-NET cell lines and normal HPNE cells. Data presented as mean ± SD.

(I) IHC staining of PIKfyve on VCaP cells treated with or without PIKfyve degrader PIK5-33d.

(J) Immunoblot analysis of GOT-1 cells showing p62 and LC3A/B expression after treatment with PIKfyve inhibitor (apilimod) for 8 or 24 hours. GAPDH was used as the loading control.

(K) Representative images of BON-1 cells treated with DMSO, apilimod, ESK981, or PIK5-33d for 8 hours (top panel). Representative images of BON-1 cells with CRISPRi-mediated NC or *PIKFYVE* knockdown (bottom panel). Scale bar: 50 µm.

(L-M) Individual tumor images of QGP-1 (L) and BON-1 (M) CDX models at the study endpoint.

Figure S2

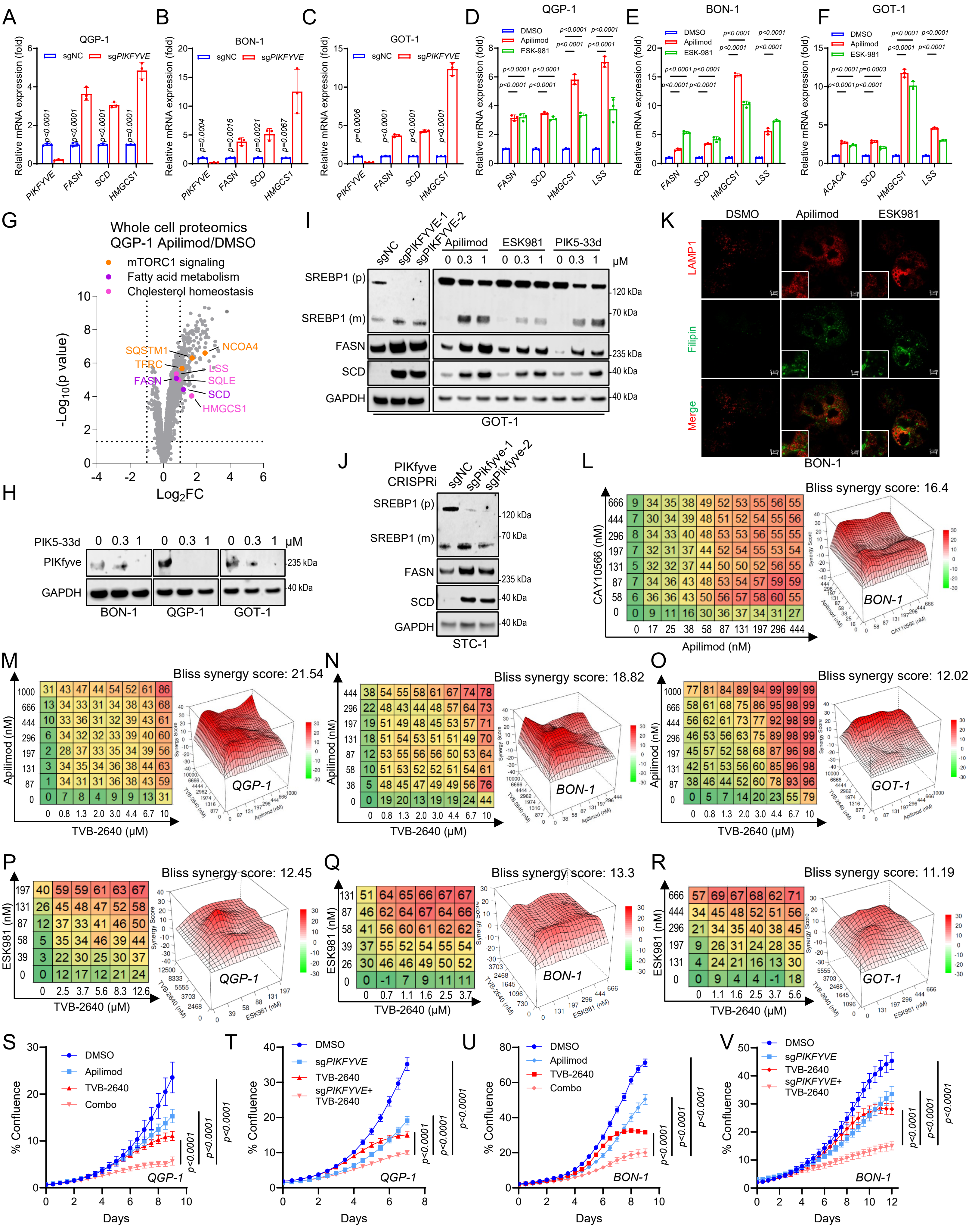

A
B
C
D
E
F
G
H
I
J
K
L
M
N
O
P
Q
R
S
T
U
V
QGP-1
BON-1
GOT-1
sgNC
sgPIKFYVE
DMSO
Apilimod
ESK-981
Relative mRNA expression (fold)
PIKFYVE
FASN
SCD
HMGCS1
LSS
ACACA
Whole cell proteomics
QGP-1 Apilimod/DMSO
mTORC1 signaling
Fatty acid metabolism
Cholesterol homeostasis
SQSTM1
NCOA4
TFRC
SQLE
-Log10(p value)
Log2FC
PIK5-33d
PIKfyve
GAPDH
sgPIKFYVE-1
sgPIKFYVE-2
SREBP1 (p)
SREBP1 (m)
120 kDa
70 kDa
235 kDa
40 kDa
μM
PIKfyve CRISPRi
sgPikfyve-1
sgPikfyve-2
STC-1
DSMO
LAMP1
Filipin
Merge
Bliss synergy score: 16.4
Bliss synergy score: 21.54
Bliss synergy score: 18.82
Bliss synergy score: 12.02
Bliss synergy score: 12.45
Bliss synergy score: 13.3
Bliss synergy score: 11.19
CAY10566 (nM)
Apilimod (nM)
TVB-2640 (μM)
ESK981 (nM)
Synergy Score
TVB-2640
Combo
sgPIKFYVE+ TVB-2640
% Confluence
Days
p<0.0001

Figure S2. PIKfyve mediates lipid homeostasis in GEP-NETs. Related to Figure 3.
(A-C) RT-qPCR of QGP-1 (A), BON-1 (B) and GOT-1 (C) cells showing changes in mRNA levels of de novo lipogenesis genes upon CRISPRi-mediated knockdown of *PIKFYVE* using sgRNA-1 targeting *PIKFYVE* compared to control. Data presented as mean ± SD (n=3). One-way ANOVA.
(D-F) RT-qPCR of QGP-1 (D), BON-1 (E) and GOT-1 (F) cells showing changes in mRNA levels of de novo lipogenesis genes following apilimod or ESK981 treatment compared to control. Data presented as mean ± SD (n=3). Two-way ANOVA.
(G) Volcano plots of differentially expressed proteins from whole cell proteomics performed on QGP-1 cells with or without apilimod treatment for 24 hours at 1 μM (specified in **Table S3**). Differentially expressed proteins involved in fatty acid metabolism (violet), cholesterol homeostasis (rose), or mTORC1 signaling pathways (orange) were highlighted with corresponding colors.
(H) Immunoblot showing PIKfyve expression in BON-1, QGP-1, and GOT-1 cells following PIKfyve degrader PIK5-33d treatment for 8 hours.
(I) Immunoblot showing premature SREBP1 (p), mature SREBP1 (m), FASN, and SCD expression in GOT-1 cells following genetic targeting or pharmacological PIKfyve inhibition (inhibitors: apilimod, ESK981; degraders: PIK5-33d. 24-hour treatment). GAPDH was used as a loading control.
(J) Immunoblot analysis of STC-1 cells showing changes in protein levels of premature or mature SREBP1, FASN, and SCD after *PIKFYVE* knockdown. Vinculin was used as a loading control.
(K) LAMP1 immunofluorescence staining with filipin (cholesterol probe) staining in BON-1 cell line showing the aggregation of free cholesterol trapped within lysosomes following apilimod or ESK981 treatment for 24 hours at 1 μM. Scale bar: 5 μm.
(L) 3D synergy plots and heatmaps for BON-1 cells treated with apilimod and CAY10566 (SCD inhibitor). The red peaks in the 3D plots (right) indicate synergistic interactions, and the overall average synergy score is shown above each plot. The heatmaps (left) depict dose-dependent decreases in cell viability for single-agent or combination treatments.
(M-O) 3D synergy plots and corresponding heatmaps for QGP-1 (M), BON-1 (N), and GOT-1 (O) cells treated with apilimod and TVB-2640 (FASN inhibitor). The red peaks in the 3D plots (right) highlight areas of synergism, with the overall average synergy score displayed above. The heatmaps (left) illustrate the reduction in cell viability across varying doses of each inhibitor, either alone or in combination.
(P-R) 3D synergy plots and heatmaps for QGP-1 (P), BON-1 (Q), and GOT-1 (R) cells treated with ESK981 and TVB-2640 (FASN inhibitor). Synergistic effects are represented by red peaks in the 3D plots (right), with the average synergy score noted above. Heatmaps (left) show cell viability reductions across different doses of each treatment, alone or in combination.
(S) Cell proliferation of QGP-1 cells showing treatment with DMSO, TVB-2640 (20 μM), apilimod (1 μM), and the combination of TVB-2640 and apilimod. Data shown are mean ± SD (n=3). Two-way ANOVA.
(T) Cell proliferation of QGP-1 cells after sgNC and sg*PIKFYVE* with or without TVB-2640. Data shown are mean ± SD (n=3). Two-way ANOVA.
(U) Confluence assay data showing the synergistic efficacy between TVB-2640 (10 μM) and apilimod (0.3 μM) condition in BON-1 cells. Data shown are mean ± SD (n=3). Two-way ANOVA.
(V) Confluence assay data showing the efficacy of TVB-2640 (10 μM) upon *PIKFYVE* knockdown in BON-1 cells. Data shown are mean ± SD (n=3). Two-way ANOVA.

Figure S3

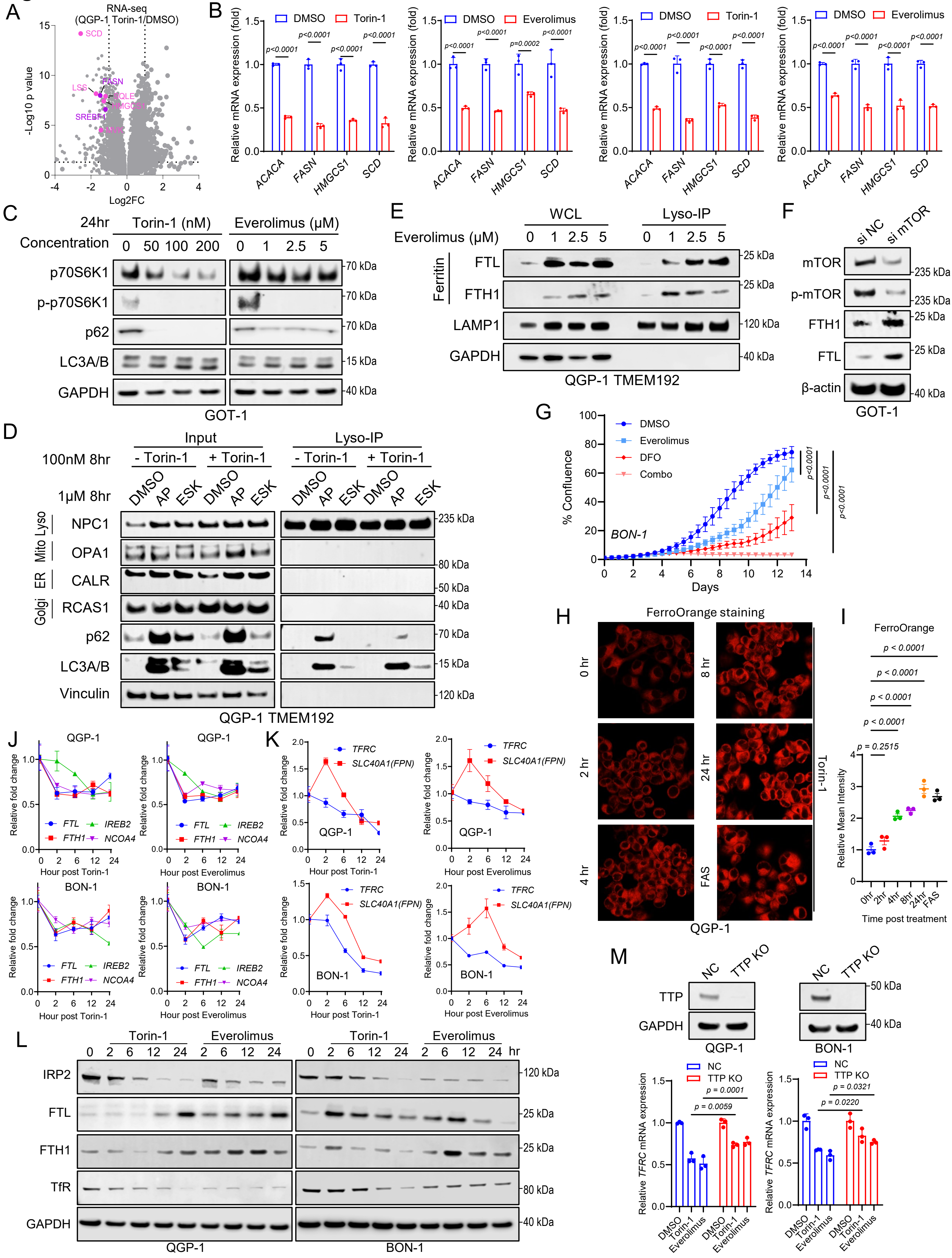

A
RNA-seq (QGP-1 Torin-1/DMSO)
-Log10 p value
Log2FC
SCD
LSS
FASN
SQLE
HMGCS1
SREBF1
MVK
B
QGP-1
BON-1
DMSO
Torin-1
Everolimus
Relative mRNA expression (fold)
ACACA
FASN
HMGCS1
SCD
C
24hr
Torin-1 (nM)
Everolimus (μM)
Concentration
p70S6K1
p-p70S6K1
p62
LC3A/B
GAPDH
GOT-1
D
100nM 8hr
Input
Lyso-IP
- Torin-1
+ Torin-1
1μM 8hr
DMSO
AP
ESK
Lyso
Mito
ER
Golgi
NPC1
OPA1
CALR
RCAS1
p62
LC3A/B
Vinculin
QGP-1 TMEM192
E
Everolimus (μM)
WCL
Lyso-IP
Ferritin
FTL
FTH1
LAMP1
GAPDH
QGP-1 TMEM192
F
si NC
si mTOR
mTOR
p-mTOR
FTH1
FTL
β-actin
GOT-1
G
% Confluence
Days
DMSO
Everolimus
DFO
Combo
BON-1
H
FerroOrange staining
0 hr
2 hr
4 hr
8 hr
24 hr
FAS
Torin-1
QGP-1
I
FerroOrange
Relative Mean Intensity
Time post treatment
J
Relative fold change
FTL
FTH1
IREB2
NCOA4
Hour post Torin-1
Hour post Everolimus
K
TFRC
SLC40A1(FPN)
L
Torin-1
Everolimus
IRP2
FTL
FTH1
TfR
GAPDH
QGP-1
BON-1
M
NC
TTP KO
TTP
GAPDH
Relative TFRC mRNA expression
DMSO
Torin-1
Everolimus

Figure S3. Inhibition of the mTOR pathway suppresses the SREBP1 pathway and triggers ferritinophagy. Related to Figure 4.

(A) Volcano plot showing differentially expressed genes of QGP-1 cells with or without Torin-1 treatment (0.1 μM, 8 hours). Genes related to enriched pathways including fatty acid metabolism (violet), cholesterol homeostasis (rose), or mTORC1 signaling pathways (orange) are labeled in the figure.

(B) RT-qPCR of indicated targets in de novo lipogenesis from QGP-1 and BON-1 cells following treatment with 0.1 μM Torin-1 or 5 μM everolimus for 8 hours. Data shown are mean ± SD (n=3). One-way ANOVA.

(C) Immunoblot analysis of phosphorylated and total p70, along with autophagy-related proteins (p62 and LC3A/B), in GOT-1 cells treated with Torin-1 or everolimus for 24 hours. GAPDH was used as the loading control.

(D) Immunoblot of input and lysosomal fractions from QGP-1 cells expressing TMEM192, treated with DMSO, apilimod (AP), ESK981 (ESK), or the combination with Torin-1. The indicated proteins were analyzed to assess lysosomal changes.

(E) Immunoblot validating increased ferritin levels (light chain: FTL, heavy chain: FTH1) observed in lysosomal proteomics of QGP-1 TMEM192 cells following everolimus treatment for 24 hours. GAPDH was used as a loading control for whole-cell lysates, while LAMP1 served as a loading control for lysosomal samples.

(F) Immunoblot showing changes in phosphorylated and total mTOR, FTH1, and FTL levels in GOT-1 cells after siRNA-mediated knockdown of *mTOR*. β-actin was used as a loading control.

(G) Confluence assay demonstrating the effect of everolimus treatment combined with iron deprivation using deferoxamine (DFO) on BON-1 cell growth. Data are shown as mean ± SD (n=4). Statistical analysis: Two-way ANOVA.

(H) Representative image of QGP-1 cells stained with FerroOrange iron dye to visualize intracellular iron following treatment with the indicated compounds for the indicated time. FAS was used as a positive control. Torin-1 was used at 100 nM, and FAS was used at 100μM. Scale bars: 20 μm.

(I) Dot plots showing normalized intracellular iron levels from panel (H). Statistical analysis: One-way ANOVA.

(J) Time-course of *FTL*, *FTH1*, *IREB2,* and *NCOA4* mRNA regulation by 0.1 μM Torin-1 (left) or 1 μM everolimus in QGP-1 (top) or BON-1 (bottom) cells.

(K) Time-course of *TFRC* and *SCL40A1* mRNA regulation by 0.1 μM Torin-1 (left) or 1 μM everolimus in QGP-1 (top) or BON-1 (bottom).

(L) Time-course of IRP2, ferritin (FTH1 and FTL), and TfR protein levels by 0.1 μM Torin-1 or 1 μM everolimus treatment in QGP-1 and BON-1 cells.

(M) Top: validation of TTP knockout in QGP-1 and BON-1 cells. Bottom: relative mRNA levels of *TFRC* in QGP-1 (left) or BON-1 (right) cells after TTP knockout treated with DMSO, 0.1 μM Torin-1, or 1 μM everolimus. Data are shown as mean ± SD (n=3). Statistical analysis: Two-way ANOVA.

Figure S4

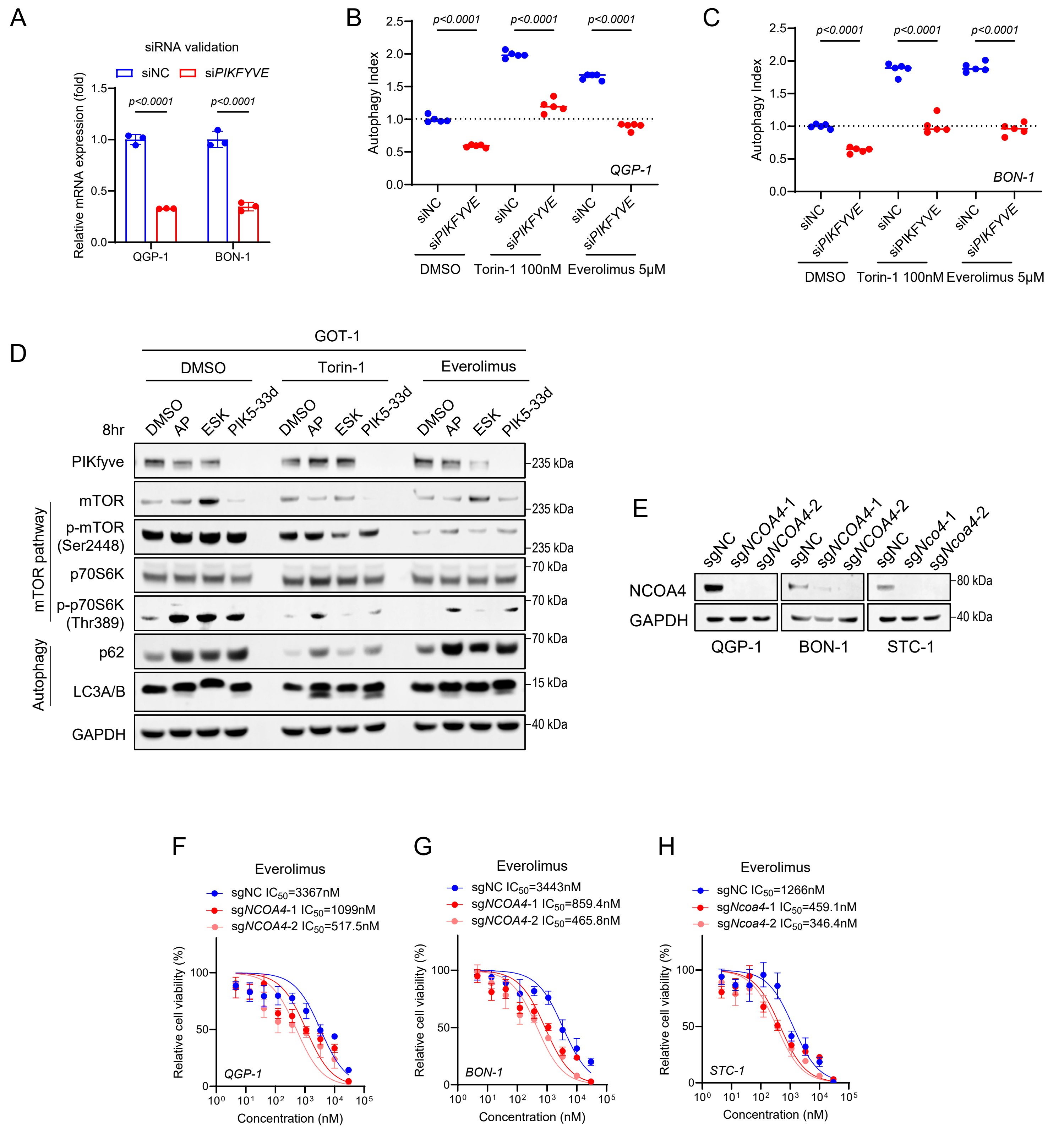

A
siRNA validation
siNC
siPIKFYVE
p<0.0001
p<0.0001
Relative mRNA expression (fold)
QGP-1
BON-1
B
p<0.0001
p<0.0001
p<0.0001
Autophagy Index
QGP-1
siNC
siPIKFYVE
DMSO
Torin-1 100nM
Everolimus 5μM
C
p<0.0001
p<0.0001
p<0.0001
Autophagy Index
BON-1
siNC
siPIKFYVE
DMSO
Torin-1 100nM
Everolimus 5μM
D
GOT-1
DMSO
Torin-1
Everolimus
8hr
DMSO
AP
ESK
PIK5-33d
PIKfyve
mTOR
p-mTOR (Ser2448)
p70S6K
p-p70S6K (Thr389)
p62
LC3A/B
GAPDH
mTOR pathway
Autophagy
235 kDa
235 kDa
235 kDa
70 kDa
70 kDa
70 kDa
15 kDa
40 kDa
E
sgNC
sgNCOA4-1
sgNCOA4-2
sgNC
sgNCOA4-1
sgNCOA4-2
sgNC
sgNcoa4-1
sgNcoa4-2
NCOA4
GAPDH
80 kDa
40 kDa
QGP-1
BON-1
STC-1
F
Everolimus
sgNC IC50=3367nM
sgNCOA4-1 IC50=1099nM
sgNCOA4-2 IC50=517.5nM
Relative cell viability (%)
QGP-1
Concentration (nM)
G
Everolimus
sgNC IC50=3443nM
sgNCOA4-1 IC50=859.4nM
sgNCOA4-2 IC50=465.8nM
Relative cell viability (%)
BON-1
Concentration (nM)
H
Everolimus
sgNC IC50=1266nM
sgNcoa4-1 IC50=459.1nM
sgNcoa4-2 IC50=346.4nM
Relative cell viability (%)
STC-1
Concentration (nM)

Figure S4. PIKfyve blockade abrogates mTOR inhibition- induced ferritinophagy. Related to Figure 5.
(A) RT-qPCR validation of siRNA-mediated knockdown of *PIKFYVE* in the indicated GFP-LC3-RFP-LC3ΔG tandem fluorescent reporter cell lines. Autophagic flux was assessed in QGP-1 (B) and BON-1 (C) cells treated with Torin-1 (0.1 μM) or everolimus (5 μM) for 24 hours. Data shown are mean ± SD (n=3). Statistical analysis using one-way ANOVA.
(D) Immunoblot analysis of mTOR signaling (phosphorylated and total mTOR, p70S6K) and autophagy markers (p62, LC3A/B) in GOT-1 cells treated with mTOR inhibitors with or without PIKfyve antagonists. GAPDH was used as a loading control.
(E) Immunoblot analysis of the indicated GEP-NET cell lines following CRISPRi-mediated knockdown of *NCOA4*. Protein levels of NCOA4 were assessed. GAPDH served as the loading control.
(F-H) IC_{50} curves of everolimus in QGP-1 (F), BON-1 (G), and STC-1 (H) cells following CRISPRi-mediated *NCOA4* knockdown. Inset lists IC_{50} values before and after *NCOA4* knockdown. Data shown are mean ± SD (n=3).

Figure S5

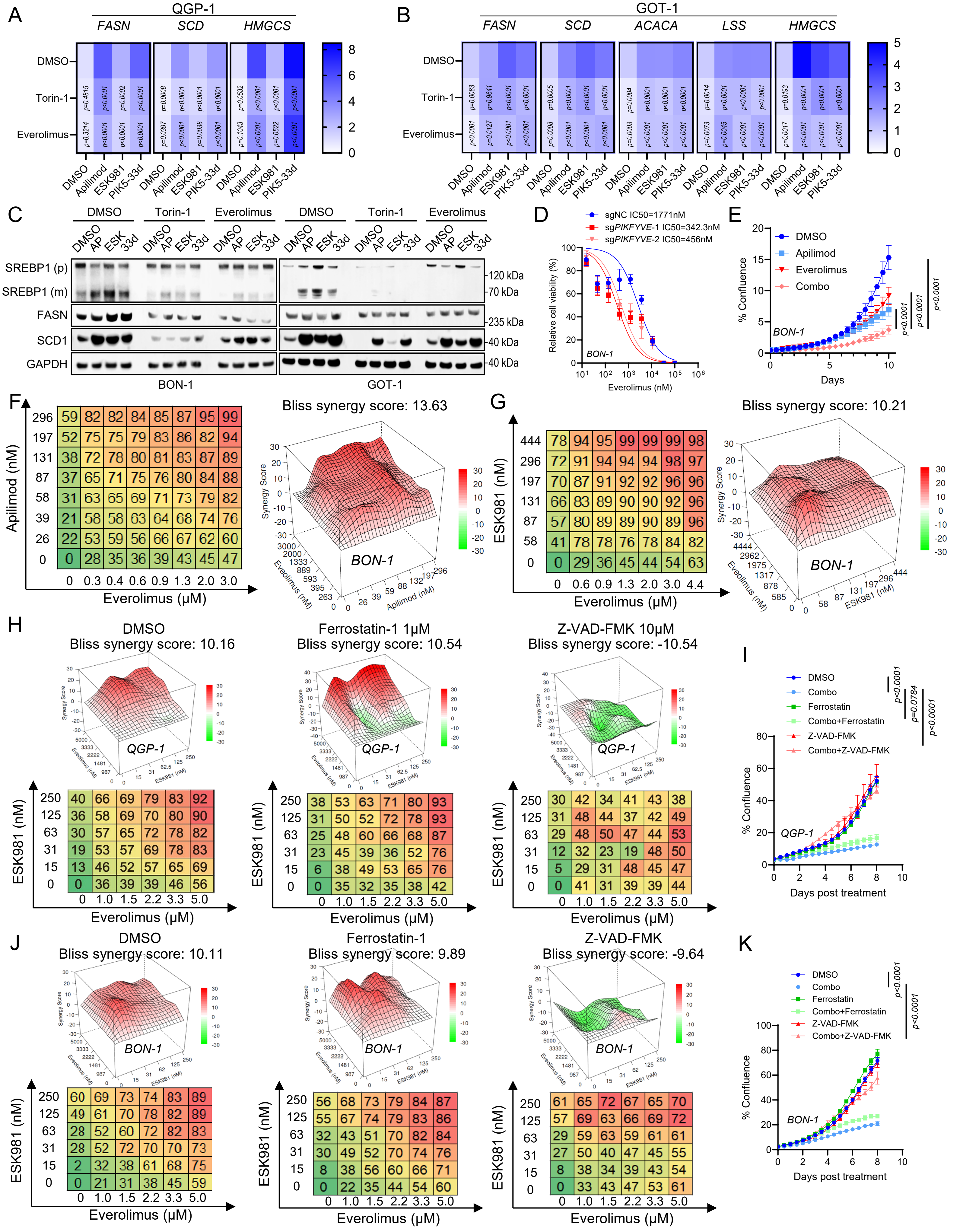
A
QGP-1
FASN
SCD
HMGCS
DMSO
Torin-1
Everolimus
Apilimod
ESK981
PIK5-33d
B
GOT-1
FASN
SCD
ACACA
LSS
HMGCS
C
SREBP1 (p)
SREBP1 (m)
FASN
SCD1
GAPDH
120 kDa
70 kDa
235 kDa
40 kDa
BON-1
GOT-1
D
sgNC IC50=1771nM
sgPIKFYVE-1 IC50=342.3nM
sgPIKFYVE-2 IC50=456nM
Relative cell viability (%)
Everolimus (nM)
BON-1
E
DMSO
Apilimod
Everolimus
Combo
% Confluence
Days
BON-1
F
Apilimod (nM)
Everolimus (µM)
Bliss synergy score: 13.63
Synergy Score
BON-1
G
ESK981 (nM)
Everolimus (µM)
Bliss synergy score: 10.21
BON-1
H
DMSO
Bliss synergy score: 10.16
Ferrostatin-1 1µM
Bliss synergy score: 10.54
Z-VAD-FMK 10µM
Bliss synergy score: -10.54
QGP-1
ESK981 (nM)
Everolimus (µM)
I
DMSO
Combo
Ferrostatin
Combo+Ferrostatin
Z-VAD-FMK
Combo+Z-VAD-FMK
p<0.0001
p=0.0784
% Confluence
Days post treatment
QGP-1
J
DMSO
Bliss synergy score: 10.11
Ferrostatin-1
Bliss synergy score: 9.89
Z-VAD-FMK
Bliss synergy score: -9.64
BON-1
ESK981 (nM)
Everolimus (µM)
K
DMSO
Combo
Ferrostatin
Combo+Ferrostatin
Z-VAD-FMK
Combo+Z-VAD-FMK
p<0.0001
% Confluence
Days post treatment
BON-1

Figure S5. Dual inhibition of mTOR and PIKfyve triggers synthetic lethality *in vitro* in GEP-NETs. Related to Figure 6.

(A-B) RT-qPCR analysis of lipid metabolism targets in QGP-1 (A) and GOT-1 (B) cells treated with mTOR inhibitors with or without PIKfyve antagonists. β-actin served as a loading control. Statistical analysis using two-way ANOVA.

(C) Immunoblot analysis of lipid metabolism in BON-1 and GOT-1 cells treated with mTOR inhibitors with or without PIKfyve antagonists. GAPDH served as a loading control.

(D) IC_{50} curves for everolimus in BON-1 cells following CRISPRi-mediated *PIKFYVE* knockdown. Inset lists IC_{50} values before and after *PIKFYVE* knockdown. Data shown are mean ± SD (n=3).

(E) Confluence assay data showing synergistic effect of apilimod (1 µM) and everolimus (5 µM) in BON-1 cells. Data shown are mean ± SEM (n=3). Two-way ANOVA.

(F-G) 3D synergy plots and heatmaps for BON-1 cells treated with everolimus and apilimod (F) or ESK981 (G). Synergistic effects are represented by red peaks in the 3D plots (right), with the average synergy score noted above. Heatmaps (left) show cell viability reductions across different doses of each treatment, alone or in combination.

(H) 3D synergy plots and heatmaps for QGP-1 cells treated with everolimus and ESK981, rescued with DMSO (left), 1 µM Ferrostatin-1 (middle), or 10 µM Z-VAD-FMK (right). Synergistic effects are represented by red peaks in the 3D plots (up), with the average synergy score noted above. Heatmaps (bottom) show cell viability reductions across different doses of each treatment, alone or in combination.

(I) Confluence assay showing synergistic effect of ESK981 (250 nM) with everolimus (5 µM) on QGP-1 cell growth rescued with DMSO, 1 µM Ferrostatin-1, or 10 µM Z-VAD-FMK treatment. Data presented as mean ± SD (n=3). Statistical analysis using two-way ANOVA.

(J) 3D synergy plots and heatmaps for BON-1 cells treated with everolimus and ESK981, rescued with DMSO (left), 1 µM Ferrostatin-1 (middle), or 10 µM Z-VAD-FMK (right). Synergistic effects are represented by red peaks in the 3D plots (up), with the average synergy score noted above. Heatmaps (bottom) show cell viability reductions across different doses of each treatment, alone or in combination.

(K) Confluence assay showing synergistic effect of ESK981 (125 nM) with everolimus (5 µM) on BON-1 cell growth rescued with DMSO, 1 µM Ferrostatin-1, or 10 µM Z-VAD-FMK treatment. Data presented as mean ± SD (n=3). Statistical analysis using two-way ANOVA.

Figure S6

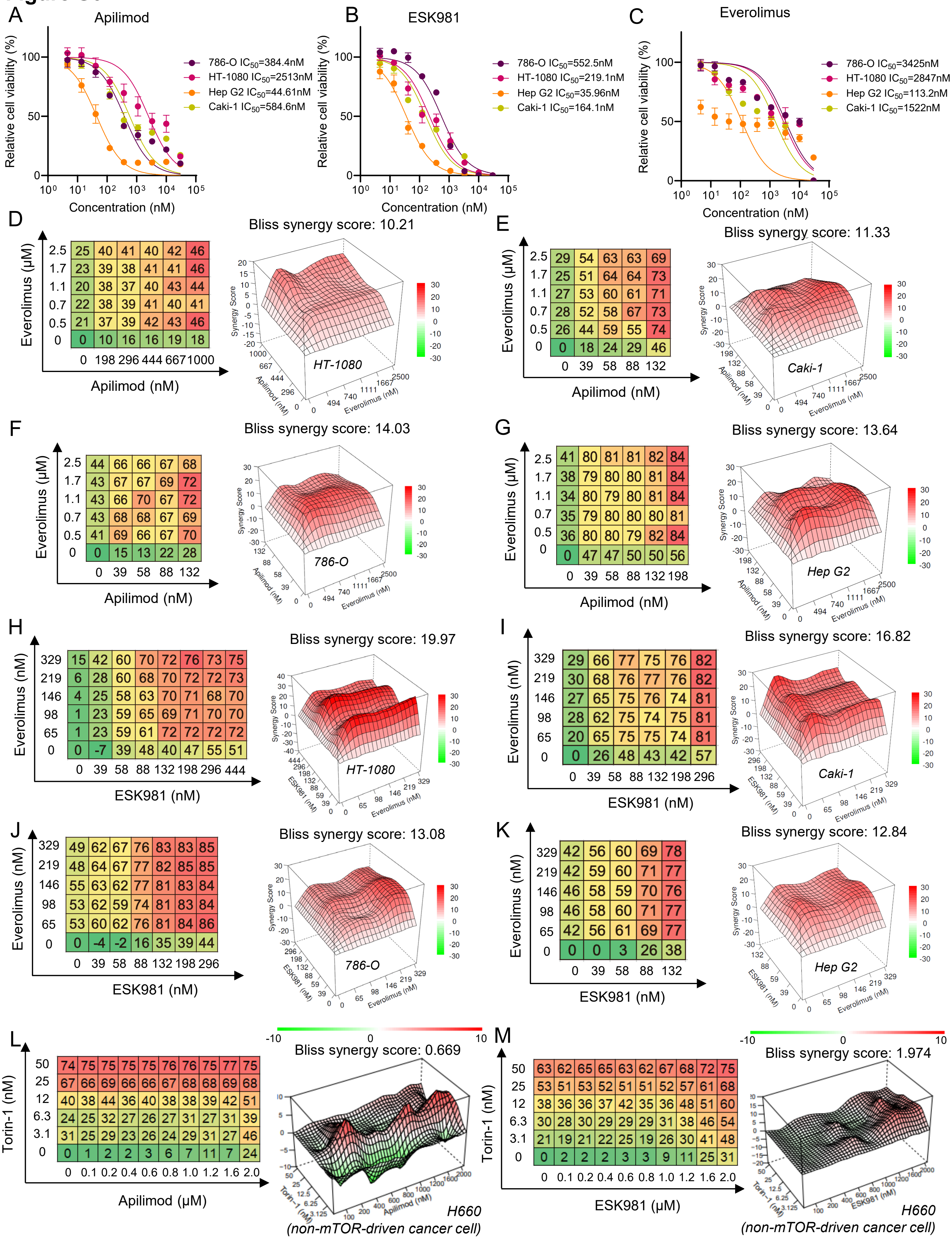

A
Apilimod
786-O IC50=384.4nM
HT-1080 IC50=2513nM
Hep G2 IC50=44.61nM
Caki-1 IC50=584.6nM
Relative cell viability (%)
Concentration (nM)
B
ESK981
786-O IC50=552.5nM
HT-1080 IC50=219.1nM
Hep G2 IC50=35.96nM
Caki-1 IC50=164.1nM
C
Everolimus
786-O IC50=3425nM
HT-1080 IC50=2847nM
Hep G2 IC50=113.2nM
Caki-1 IC50=1522nM
D
Bliss synergy score: 10.21
Everolimus (μM)
2.5 25 40 41 40 42 46
1.7 23 39 38 41 41 46
1.1 20 38 37 40 43 44
0.7 22 38 39 41 40 41
0.5 21 37 39 42 43 46
0 0 10 16 16 19 18
0 198 296 444 667 1000
Apilimod (nM)
HT-1080
Synergy Score
Everolimus (nM)
E
Bliss synergy score: 11.33
2.5 29 54 63 63 69
1.7 25 51 64 64 73
1.1 27 53 60 61 71
0.7 28 52 58 67 73
0.5 26 44 59 55 74
0 0 18 24 29 46
0 39 58 88 132
Apilimod (nM)
Caki-1
F
Bliss synergy score: 14.03
2.5 44 66 66 67 68
1.7 43 67 67 69 72
1.1 43 66 70 67 72
0.7 43 68 68 67 69
0.5 41 69 66 67 70
0 0 15 13 22 28
0 39 58 88 132
Apilimod (nM)
786-O
G
Bliss synergy score: 13.64
2.5 41 80 81 81 82 84
1.7 38 79 80 80 81 84
1.1 34 80 79 80 81 84
0.7 35 79 80 80 80 81
0.5 36 80 80 79 82 84
0 0 47 47 50 50 56
0 39 58 88 132 198
Apilimod (nM)
Hep G2
H
Bliss synergy score: 19.97
Everolimus (nM)
329 15 42 60 70 72 76 73 75
219 6 28 60 68 70 72 72 73
146 4 25 58 63 70 71 68 70
98 1 23 59 65 69 71 70 70
65 1 23 59 61 72 72 72 72
0 0 -7 39 48 40 47 55 51
0 39 58 88 132 198 296 444
ESK981 (nM)
HT-1080
I
Bliss synergy score: 16.82
329 29 66 77 75 76 82
219 30 68 76 77 76 82
146 27 65 75 76 74 81
98 28 62 75 74 75 81
65 20 65 75 75 74 81
0 0 26 48 43 42 57
0 39 58 88 132 198 296
ESK981 (nM)
Caki-1
J
Bliss synergy score: 13.08
329 49 62 67 76 83 83 85
219 48 64 67 77 82 85 85
146 55 63 62 77 81 83 84
98 53 62 59 74 81 83 84
65 53 60 62 76 81 84 86
0 0 -4 -2 16 35 39 44
0 39 58 88 132 198 296
ESK981 (nM)
786-O
K
Bliss synergy score: 12.84
329 42 56 60 69 78
219 42 59 60 71 77
146 46 58 59 70 76
98 46 58 60 71 77
65 42 56 61 69 77
0 0 0 3 26 38
0 39 58 88 132
ESK981 (nM)
Hep G2
L
Bliss synergy score: 0.669
Torin-1 (nM)
50 74 75 75 75 75 76 76 75 77 75
25 67 66 69 66 66 67 68 68 69 68
12 40 38 44 36 40 38 38 39 42 51
6.3 24 25 32 27 26 27 31 27 31 39
3.1 31 25 29 23 26 24 29 31 27 46
0 0 1 2 2 3 6 7 11 7 24
0 0.1 0.2 0.4 0.6 0.8 1.0 1.2 1.6 2.0
Apilimod (μM)
H660
(non-mTOR-driven cancer cell)
M
Bliss synergy score: 1.974
50 63 62 65 65 63 62 67 68 72 75
25 53 51 53 52 51 51 52 57 61 68
12 38 36 36 37 42 35 36 48 51 60
6.3 30 28 30 29 29 29 31 38 46 54
3.1 21 19 21 22 25 19 26 30 41 48
0 0 2 2 2 3 3 9 11 25 31
0 0.1 0.2 0.4 0.6 0.8 1.0 1.2 1.6 2.0
ESK981 (μM)
H660
(non-mTOR-driven cancer cell)

Figure S6. PIKfyve and mTOR inhibition synergize to decrease cell growth in mTOR-driven cancer cell lines. Related to Figure 6.

(A-C) Dose-dependent cell growth inhibition of apilimod (A), ESK981(B), or everolimus (C) in the indicated mTOR-driven cancer cell lines. Data shown are mean ± SD (n=3).

(D-G) 3D synergy plots and heatmaps for HT-1080 (D), Caki-1(E), 786-O (F), or Hep G2 (G) cells treated with everolimus and apilimod. Synergistic effects are represented by red peaks in the 3D plots (right), with the average synergy score noted above. Heatmaps (left) show cell viability reductions across different doses of each treatment, alone or in combination.

(H-K) 3D synergy plots and heatmaps for HT-1080 (H), Caki-1(I), 786-O (J), or Hep G2 (K) cells treated with everolimus and ESK981. Synergistic effects are represented by red peaks in the 3D plots (right), with the average synergy score noted above. Heatmaps (left) show cell viability reductions across different doses of each treatment, alone or in combination.

(L-M) 3D synergy plots and heatmaps for NCI-H660 treated with Torin-1 and apilimod (L) or Torin-1 and ESK981 (M). Synergistic effects are represented by red peaks in the 3D plots (right), with the average synergy score noted above. Heatmaps (left) show cell viability reductions across different doses of each treatment, alone or in combination.

Figure S7

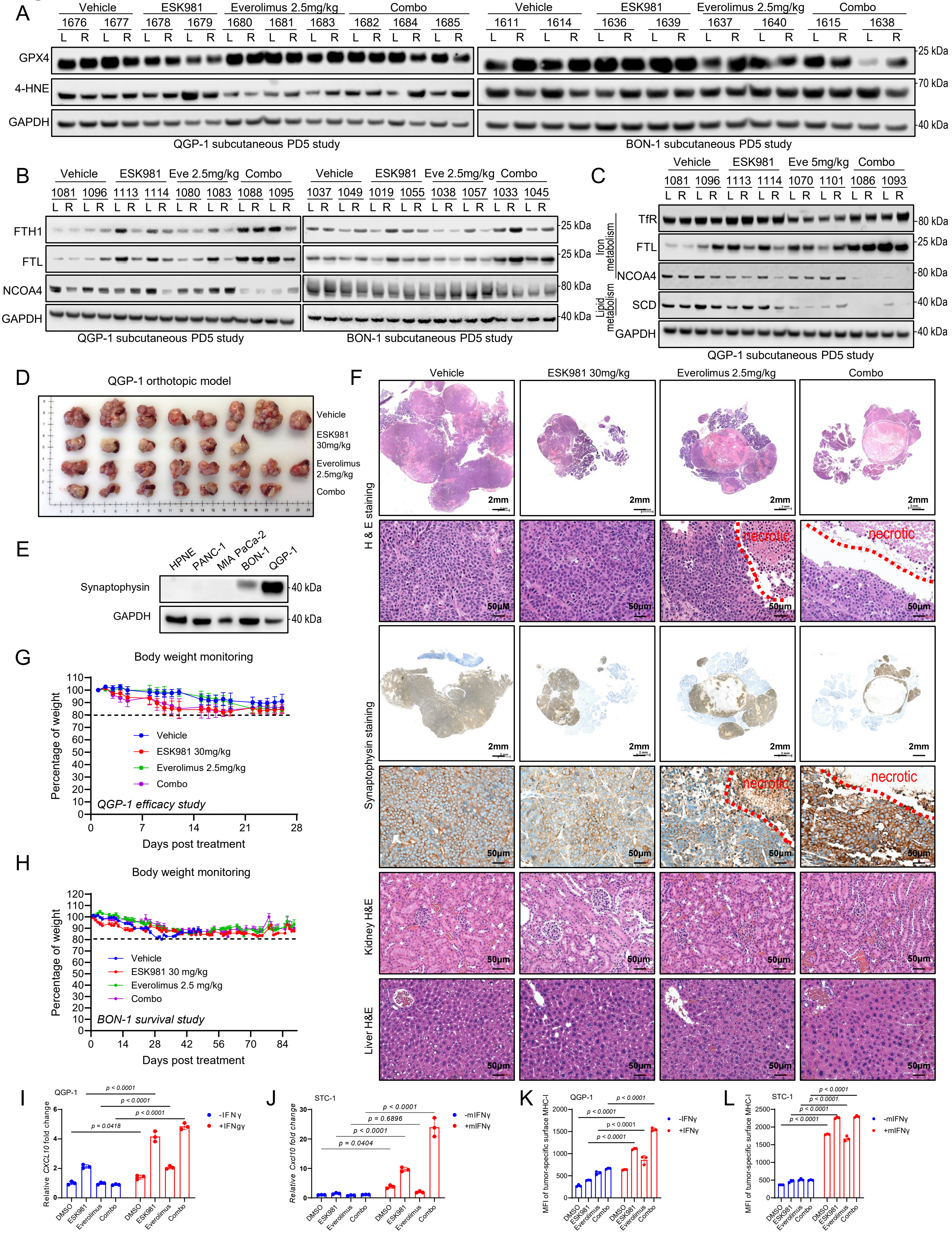
A
Vehicle
ESK981
Everolimus 2.5mg/kg
Combo
1676 1677 1678 1679 1680 1681 1683 1682 1684 1685
1611 1614 1636 1639 1637 1640 1615 1638
GPX4
4-HNE
GAPDH
25 kDa
70 kDa
40 kDa
QGP-1 subcutaneous PD5 study
BON-1 subcutaneous PD5 study
B
Eve 2.5mg/kg
FTH1
FTL
NCOA4
80 kDa
C
Eve 5mg/kg
TfR
Iron metabolism
Lipid metabolism
SCD
D
QGP-1 orthotopic model
ESK981 30mg/kg
Everolimus 2.5mg/kg
E
HPNE
PANC-1
MIA PaCa-2
BON-1
QGP-1
Synaptophysin
F
ESK981 30mg/kg
H & E staining
Synaptophysin staining
Kidney H&E
Liver H&E
2mm
50μm
necrotic
G
Body weight monitoring
Percentage of weight
QGP-1 efficacy study
Days post treatment
H
BON-1 survival study
ESK981 30 mg/kg
Everolimus 2.5 mg/kg
I
QGP-1
Relative CXCL10 fold change
p = 0.0418
p < 0.0001
-IFNγ
+IFNgγ
DMSO
Everolimus
J
STC-1
Relative Cxcl10 fold change
p = 0.0404
p = 0.6896
-mIFNγ
+mIFNγ
K
MFI of tumor-specific surface MHC-I
-IFNγ
+IFNγ
L

Figure S7. Combinatorial targeting of mTOR and PIKfyve exerts synergistic effects *in vivo* in GEP-NETs. Related to Figure 7.

(A) Immunoblot analysis of QGP-1 and BON-1 CDX tumors after 5 days (PD5) treatment with vehicle, ESK981 (30 mg/kg), everolimus (2.5 mg/kg), or the combination, showing protein levels of GPX4 and 4-hydroxynonenaL (4-HNE). GAPDH served as a loading control.

(B) Immunoblot analysis of QGP-1 and BON-1 CDX tumors after 5 days (PD5) treatment with vehicle, ESK981 (30 mg/kg), everolimus (2.5 mg/kg), or the combination, showing protein levels of ferritin (FTH1 and FTL) and NCOA4. GAPDH served as a loading control.

(C) Immunoblot analysis of QGP-1 CDX tumors after 5 days (PD5) treatment with vehicle, ESK981 (30 mg/kg), everolimus (5 mg/kg), or the combination, showing levels of proteins involved in the iron metabolism pathway (TfR, FTL, and NCOA4) and lipid metabolism pathway (SCD). GAPDH served as a loading control.

(D) Image of individual pancreas and tumors from QGP-1 orthotopic model shown in Figure 7C.

(E) Immunoblot analysis of synaptophysin expression in pancreatic duct cells (HPNE), adenocarcinoma cells (PANC-1, MIA PaCa-2), and neuroendocrine tumor cells (BON-1, QGP-1). GAPDH served as the loading control.

(F) H&E and synaptophysin IHC staining for Figure 7D showing tumor area across the whole pancreas region. H&E staining for Figure 7D showing histological structure in kidney and liver. Scalebars for each row are labelled.

(G) Percentage body weight changes of QGP-1 tumor bearing mice from indicated treatment groups in Figure 7C. Data shown are mean ± SD (n=3).

(H) Percentage body weight changes of BON-1 tumor bearing mice from indicated treatment groups in Figure 7H. Data shown are mean ± SD (n=3).

(I-J) *CXCL10* mRNA levels of QGP-1 (I) or STC-1 (J) cells stimulated with or without IFN-γ at 10 ng/mL and treated with the indicated agents for 24 h. ESK981 was used at 1 μM for QGP-1 and 0.5 μM for STC-1. Everolimus was used at 1 μM for both. Data shown are mean ± SD (n=3). Statistical analysis using two-way ANOVA.

(K-L) Quantification of flow cytometry measuring MHC-I surface expression in QGP-1 (K) or STC-1 (L) cells stimulated with or without IFN-γ at 10 ng/mL and treated with the indicated agents for 24 h. ESK981 was used at 1 μM for QGP-1 and 0.5 μM for STC-1. Everolimus was used at 1 μM for both. Data shown are mean ± SD (n=3). Statistical analysis using two-way ANOVA.
